# Use of a combined antibacterial synergy approach and the ANNOgesic tool to identify novel targets within the gene networks of multidrug-resistant *Klebsiella pneumoniae*

**DOI:** 10.1128/msystems.00877-23

**Published:** 2024-02-13

**Authors:** Hyunsook Lee, Sung-Huan Yu, Jung Eun Shim, Dongeun Yong

**Affiliations:** 1 Department of Laboratory Medicine and Research Institute of Bacterial Resistance, Yonsei University College of Medicine, Seoul, South Korea; 2 Brain Korea 21 PLUS Project for Medical Science, Yonsei University College of Medicine, Seoul, South Korea; 3 Institute of Precision Medicine, College of Medicine, National Sun Yat-sen University, Kaohsiung, Taiwan; 4 School of Medicine, College of Medicine, National Sun Yat-sen University, Kaohsiung, Taiwan; 5 Bioinformatics Collaboration Unit, Yonsei Biomedical Research Institute, Yonsei University College of Medicine, Seoul, South Korea; LifeMine Therapeutics, Cambridge, Massachusetts, USA

**Keywords:** multidrug-resistant (MDR), meropenem, colistin, chemical compound, synergy, RNA sequencing, ANNOgesic, noncoding RNA, gene network

## Abstract

Since the 1980s, the development of new drug classes for the treatment of multidrug-resistant *Klebsiella pneumoniae* has become limited, highlighting the urgent need for novel antibiotics. To address this challenge, this study aimed to explore the synergistic interactions between chemical compounds and representative antibiotics, such as carbapenem and colistin. The primary objective of this study was not only to mitigate the adverse impact of multidrug-resistant *K. pneumoniae* on public health but also to establish a sustainable balance among humans, animals, and the environment. Phenotypical measurements were conducted using the broth microdilution technique to determine the drug sensitivity of bacterial strains. Additionally, a genotypical approach was employed, involving traditional RNA sequencing analysis to identify differentially expressed genes and the computational ANNOgesic tool to detect noncoding RNAs. This study revealed the existence of various pathways and regulatory RNA elements that form a functional network. These pathways, characterized by the expression of specific genes, contribute to the combined treatment effect and bacterial survival strategies. The connections between pathways are facilitated by regulatory RNA elements that respond to environmental changes. These findings suggest an adaptive response of bacteria to harsh environmental conditions.

**IMPORTANCE:**

Noncoding RNAs were identified as key players in post-transcriptional regulation. Moreover, this study predicted the presence of novel small regulatory RNAs that interact with target genes, as well as the involvement of riboswitches and RNA thermometers in conjunction with associated genes. These findings will contribute to the discovery of potential antimicrobial therapeutic candidates. Overall, this study offers valuable insights into the synergistic effects of chemical compounds and antibiotics, highlighting the role of regulatory RNA elements in bacterial response, and survival strategies. The identification of novel noncoding RNAs and their interactions with target genes, riboswitches, and RNA thermometers holds promise for the development of antimicrobial therapies.

## INTRODUCTION

The escalating mortality attributed to antibiotic resistance has surpassed that of other major diseases, rendering it a critical global clinical concern (1). Bacterial evolution has outpaced the emergence of Food and Drug Administration-approved antibiotics, intensifying the antibiotic resistance crisis and causing significant economic losses in the healthcare industry and healthcare systems.

Gram-negative *Klebsiella pneumoniae*, categorized as an ESKAPE opportunistic pathogen, poses a threat via both nosocomial and community-acquired infections (2). Among these, Sequence Type 11 K56 is a multidrug-resistant strain that lacks carbapenemase production (3), and no colistin-related resistance genes have been detected at the DNA level (Table S1) (4). K56 shares a vertical gene transfer relationship with K26 and is susceptible to both meropenem and colistin. K56 also exhibits adaptive resistance under stress exposure, such as harsh environmental conditions (5).

Meropenem, a broad-spectrum β-lactam antibiotic, covalently binds to penicillin-binding proteins and disrupts cell wall synthesis. Colistin, a polymyxin E antibiotic, interacts with lipopolysaccharides and phospholipids, leading to outer membrane disruption, increased cell permeability, and subsequent cell lysis and death (6). However, colistin monotherapy often causes adverse effects, such as nephrotoxicity and neurotoxicity (7). The combination of colistin with existing antibiotics presents a potential solution against multidrug-resistant Gram-negative bacteria (8).

Previous studies have investigated the properties and mechanisms of K56 in response to meropenem treatment while also exploring colistin-related resistance mechanisms (3). Through transcriptome analysis, complementation, and knockout techniques, these studies have identified both agonists (downregulated genes) and antagonists (upregulated genes) associated with meropenem treatment. However, certain barriers have hindered the ability to create new drugs and selectively knock out genes for therapeutic use.

Combination therapy is emerging as a strategy to minimize the detrimental impact of diseases on global public health by preventing transmission among humans, animals, and the environment. Repurposing or partially repurposing existing drugs offers a novel approach with a broad spectrum of action, reduced likelihood of development of resistance compared with monotherapy, and effectiveness against diverse strains (9). Moreover, the use of low therapeutic doses further minimizes side effects while maintaining practical efficacy.

In addition to the phenotypic approach, a genotypic approach can be employed to unveil the hidden aspects of the K56 response. Transcriptome analysis has enabled the discovery of differentially expressed genes (DEGs) involved in antibiotic resistance and therapeutic mechanisms. For extensive investigation, ANNOgesic was used to analyze the characteristics of K56 after exposure to combination treatment. Many of the genome annotation tools are sequence-based, such as Prokka and EuGene-PP, but only using a reference genome cannot reflect the real situations of different experimental conditions. ANNOgesic is a software that can identify more than 20 bacterial genomic features based on RNA-Seq (10). This modular tool also optimizes parameters and removes false positives for high-quality results (11). Affected genes can be identified based on ANNOgesic analysis of a variety of regulatory RNAs, including RNA thermometers, small regulatory RNAs (sRNAs), and riboswitches. These regulatory RNAs have exhibited diverse roles in physiological functions, developmental metabolism, and virulent pathogenesis (12), including enhancing the efficacy of combination treatment and reducing fitness under limited conditions to combat antibiotic resistance (13).

## RESULTS

### K56’s survival strategy against chemical #3 single treatment

**Fig 1:**
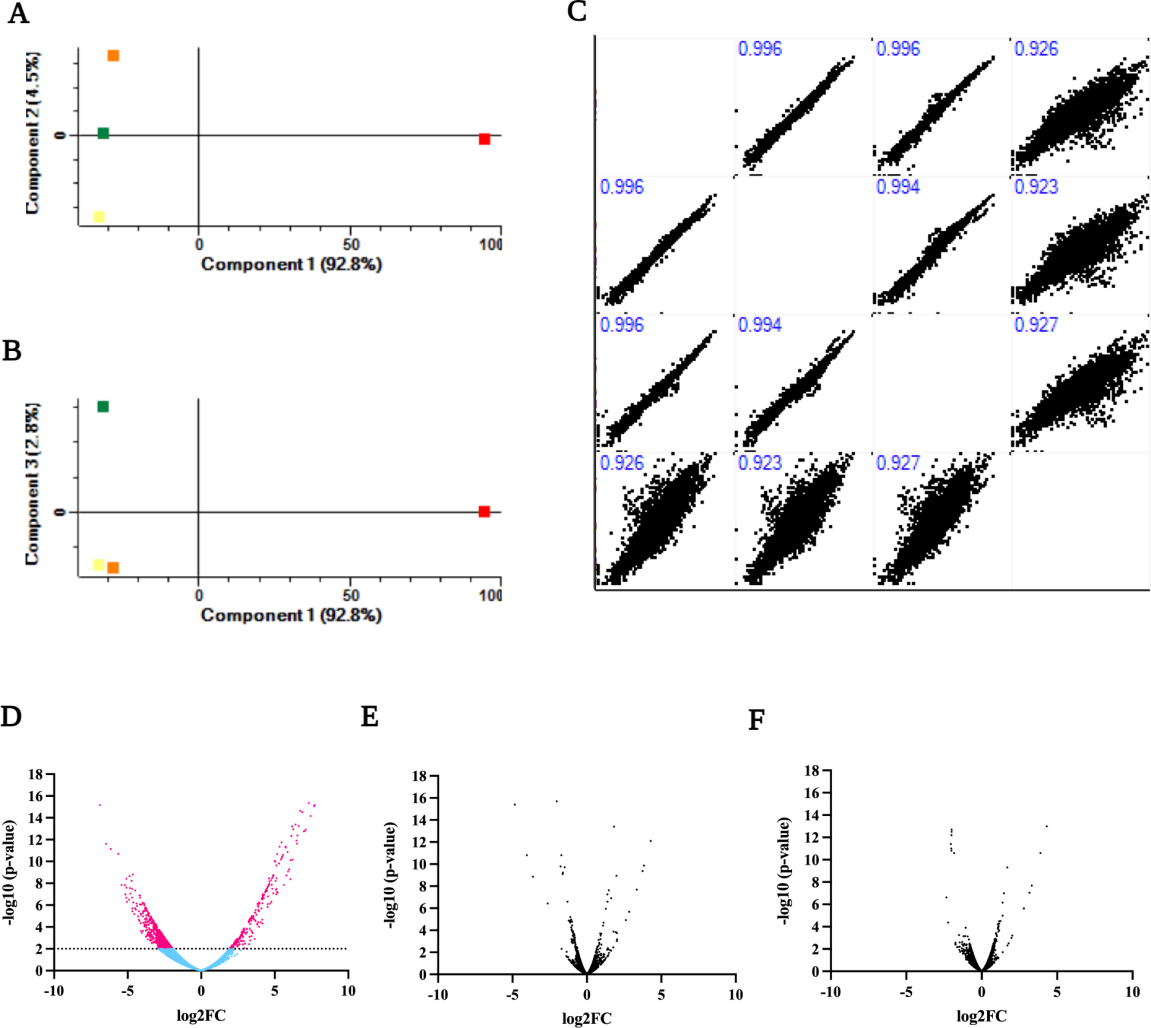
The effect of combination treatment (K56_Co_Che) is shown in correlation and differential expression plots. Principal component analysis was performed. Red, orange, yellow, and green squares represent the K56_Co_Che, K56_Che, K56_Co, and K56 (control) groups, respectively. Panel (**A**) shows the plot based on components 1 and 2, and panel (**B**) displays the results of components 1 and 3. The graphs indicate the relatively greater significance of K56_Co_Che. Multiple scatter plots with correlations are shown in panel (**C**). The plots illustrate the positive association and linear relationships between the values. The dispersion of the values reveals patterns and relationships between variables. The combination treatment frequently exhibited a wider numerical distribution than those of the two single treatments and the control. The correlation coefficient values ranged from 0.923 to 0.996, with higher values indicating closer proximity between the groups in terms of linear relationships. Volcano plots of (**D**) K56 vs K56_Co_Che, (**E**) K56 vs K56_Che, and (**F**) K56 vs K56_Co groups are shown. The X-axis represents the log2 fold change (log2FC). The Y-axis represents the −log10(*P*-value) based on the LPEseq method. Genes on the left side of the plots are downregulated, while those on the right side are upregulated. The dotted line represents the cutoff with a *P*-value of 0.01 for identifying DEGs. Magenta dots represent DEGs and blue dots represent non-DEGs. Plot (**D**) shows a higher number of expressed genes in the combination treatment condition than those in plots (**E**) and (**F**).

Among the 6,696 chemicals tested, six compounds showed synergistic with meropenem, but only chemical #3 remained an additive at a low concentration. When the meropenem was replaced by colistin, chemical #3 became synergistic. This chemical #3 group had highly similar gene expression to that of the control group, with Pearson correlation coefficients of 0.996 (Fig. 1C). In contrast to the combination treatment, the single treatment exhibited a considerably smaller number of DEGs (Fig. 1D).

As a metabolic linking mechanism of chemical #3 only treatment, the inositol catabolic process occurs. Inositol is found in the phospholipids of cell membranes (14). Inositol-1,2,3,4,5 phosphates are downregulated (Fig. S1), but there are no gene expression changes in myo-inositols. On the other hand, D-chiro-inositols are significantly decreased. The reduced inositol metabolism causes decreased carbon availability due to the nutrient-poor condition without changes in phospholipids for cell permeability. The other key pathway is the phosphoenolpyruvate-dependent sugar phosphotransferase system (PTS), which catalyzes transport and phosphorylation in bacteria (15). The MtlA (KPHS_26190 phosphoenolpyruvate-dependent PTS family enzymes IIA component) with downregulated gene expression (Fig. S1B) forms the PTS phosphorylation cascade. Thus, reduced sugar transport and phosphorylation cause an energy decline.

### Combination treatment (colistin and chemical #3) of K56 significantly affects its gene expression

Combination treatment of K56 with colistin and chemical #3 (K56_Co_Che) exhibited a substantial impact on gene expression (Fig. S2), as indicated by the principal component analysis. The K56_Co_Che group demonstrated a marked difference compared to the other treatment groups and the control group (Fig. 1; Fig. S3), which was further supported by correlation analysis (Fig. 1). To identify the factors that influence gene expression, a differential expression analysis was conducted to investigate significantly DEGs (Table S2). The volcano plot revealed 242 upregulated and 580 downregulated genes with a *P*-value of 0.01. Almost no overlap in DEGs was observed among the three treatment groups (Fig. S4).

### Global downregulation of phenylalanine metabolism

**Fig 2:**
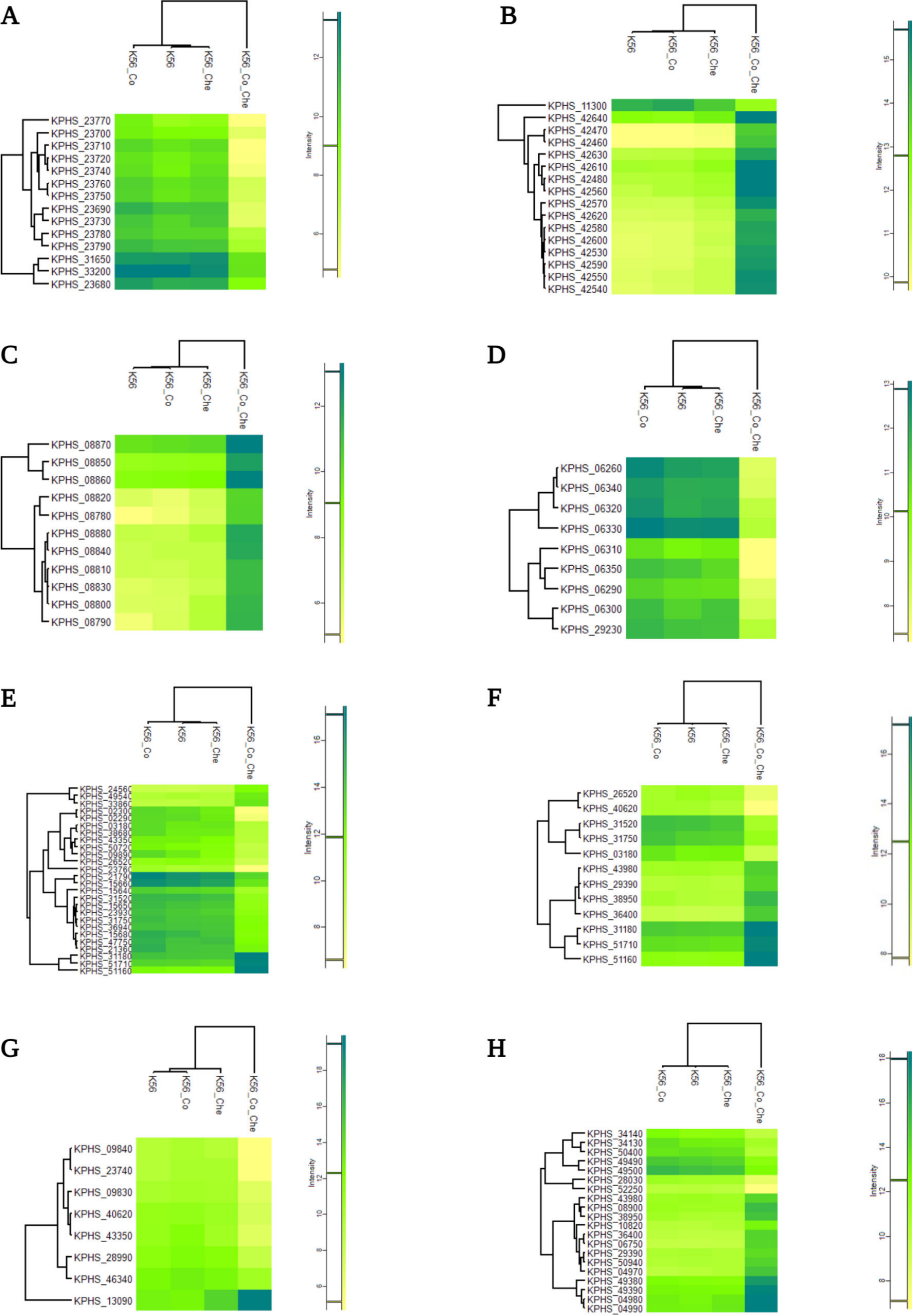
Heatmaps of various pathways. (**A**) In phenylalanine metabolism, KPHS_23690 and KPHS_23710, which were annotated as phenylacetate-CoA oxygenase subunit PaaA and phenylacetic acid degradation protein, were markedly downregulated. (**B**) Under the K56_Co_Che combination treatment, porphyrin metabolism genes were highly expressed, except for KPHS_11300 (protoheme IX farnesyltransferase). KPHS_42470 (adenosylcobinamide kinase/adenosylcobinamide-phosphate guanylyltransferase) and KPHS_42460 (nicotinate-nucleotide-dimethylbenzimidazole phosphoribosyltransferase) were strongly increased. (**C**) Type II secretion system (T2SS). Overall, gene expression of the type II secretion system showed a positive response to the combination treatment. In the colistin-only condition, KPHS_08820 (pullulanase I protein) exhibited slightly lower expression than that in the control group (K56 only). (**D**) Tyrosine metabolism. The gross pattern of tyrosine metabolism decreased under combination treatment. KPHS_06330 (4-hydroxyphenlacetate catabolism) and KPHS_06260 (4-hydroxyphenlacetate catabolism) were remarkably enhanced in the colistin-only single-treatment condition. Both genes participated in 4-hydroxypenylacetate catabolism, including phenylacetate breakdown. (**E**) Carbon metabolism. Expression of KPHS_02300 (isocitrate lyase), KPHS_02290 (malate synthase), and KPHS_23760 (3-hydroxy butyryl-CoA dehydrogenase) distinctly decreased in the K56_Co_Che combination treatment condition, whereas KPHS_31180 (pyruvate kinase), KPHS_51710 (triosephosphate isomerase), and KPHS_51160 (phosphoglyceromutase) showed enhanced gene expression under the combination treatment. In the (**F**) glycolysis or gluconeogenesis pathway, KPHS_03180 (acetyl-CoA synthetase), KPHS_26520 (glyceraldehyde-3-phosphate dehydrogenase), and KPHS_40620 (lactaldehyde dehydrogenase) expression levels were decreased compared to those in the control and single-treatment conditions. (**G**) Lysine degradation. Upregulation of KPHS_13090 (lysine decarboxylase 1) and downregulation of KPHS_09840 (glutarate 2-hydroxylase) and KPHS_23740 (enoyl-CoA hydratase-isomerase) were significantly changed after combination treatment exposure. (**H**) Starch and sucrose metabolism. KPHS_04990 (trehalose(maltose)-specific PTS system component IIBC) was upregulated and KPHS_52250 (putative PTS, EIIC) was downregulated.

**Fig 3:**
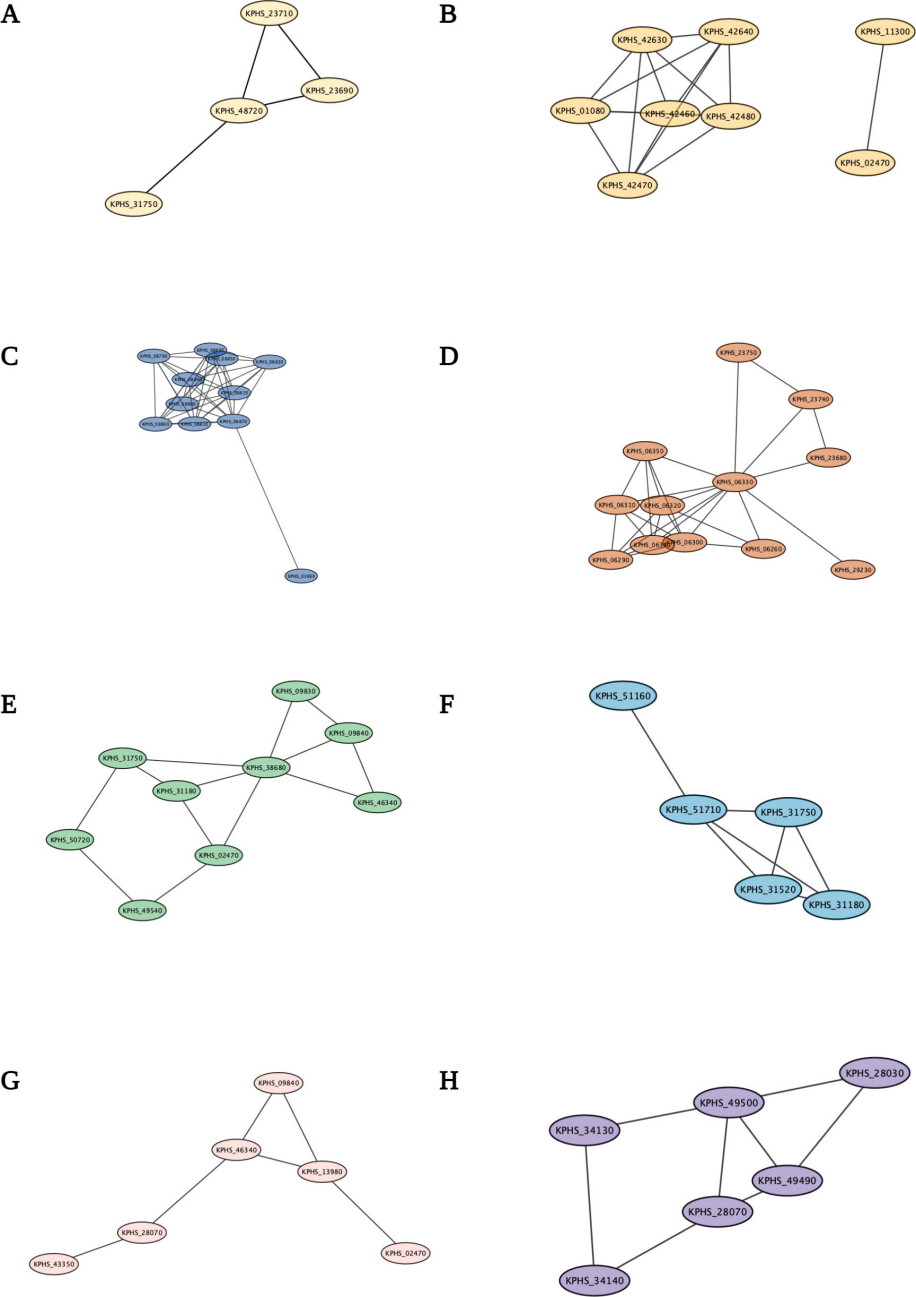
Functional networks among differentially expressed genes and noncoding RNA-associated genes. (**A**) KPHS_23690 and KPHS_23710 are associated with the downregulation of KPHS_48720 in porphyrin metabolism. KPHS_48720 is linked to KPHS_31750, which is associated with carbon metabolism, glucose, or gluconeogenesis, and the RNA thermometer. (**B**) KPHS_01080 is a vitamin B_12_ (cobalamin) outer membrane transporter that forms a network with positively expressed KPHS_42470, KPHS_42460, KPHS_42480 (cobyric acid synthase), KPHS_42640 (cobyric acid a, c-diamide synthase), and KPHS_42630 (cobalamin biosynthesis protein). These genes exhibit positive expression in the porphyrin metabolism pathway. (**C**) The differentially expressed genes are functionally connected with KPHS_01080, which is associated with the cobalamin riboswitch. (**D**) KPHS_06330, KPHS_23680 (bifunctional aldehyde dehydrogenase/enoyl-CoA hydratase), KPHS_06340 (4-hydroxyphenlacetate degradation bifunctional isomerase/decarboxylase C-terminal subunit), KPHS_06310 (4-hydroxyphenlacetate catabolism), and KPHS_06350 (4-hydroxyphenlacetate catabolism) are linked in a functional network. (**E**) According to the Database for Annotation, Visualization, and Integrated Discovery (DAVID), the downregulation of KPHS_31750 (6-phosphofructokinase) is functionally related to the downregulation of KPHS_50720 (putative kinase) and KPHS_38680 (transaldolase kinase) and the upregulation of KPHS_49540 (gluconate kinase) and KPHS_31180 (pyruvate kinase). KPHS_38680 is an enzyme involved in the pentose phosphate pathway and forms an integrative network with KPHS_02470 (glucose-6-phosphate), KPHS_09830 (hypothetical protein), KPHS_09840 (hypothetical protein), and KPHS_46340 (putrescine). Notably, a lysine riboswitch is associated with KPHS_02470. (**F**) Decreased levels of KPHS_31520 (phosphoenolpyruvate synthase) and KPHS_31750 (6-phosphofructokinase), along with increased levels of KPHS_51710 (triosephosphate isomerase), KPHS_31180 (pyruvate kinase), and KPHS_51160 (phosphoglyceromutase), form a functionally related integrative network. (**G**) KPHS_23680 (enoyl-CoA hydratase), which is involved in phenylalanine metabolism, is linked to KPHS_23740. Downregulation of KPHS_46340 (putrescine aminotransferases) and KPHS_43350 (acetyl-CoA acetyltransferase) results in a connection with the target gene KPHS_28070, which is a novel noncoding sRNA. KPHS_02470 (glucose-6-phosphate isomerase) is a positively expressed lysine riboswitch-associated gene. KPHS_02470 reduced KPHS_09840 levels, and KPHS_46340 form a network indirectly through KPHS_13980 (carboxylate-amine ligase). (**H**) The genes shown are linked to slight downregulation of both KPHS_34130 (alpha, alpha-trehalose-phosphate synthase) and KPHS_34140 (trehalose-6-phosphate phosphatase). KPHS_34130 is linked to the downregulation of KPHS_31750 and the upregulation of KPHS_31180. In particular, KPHS_34140 is directly associated with KPHS_28070 (general stress protein), the target gene of a novel sRNA predicted by the ANNOgesic analysis.

Decreased expression of the KPHS_23690 (phenylacetate-CoA oxygenase subunit PaaA) and KPHS_23710 (phenylacetic acid degradation protein) genes leads to reduced succinyl-CoA and acetyl-CoA levels (16). This decrease in gene expression negatively affects the citrate cycle, as observed in the metabolic pathway analysis results (Fig. 2A). Furthermore, these two genes were functionally linked to the downregulation of KPHS_48720 (bacterioferritin). The iron ion transport system was related to the porphyrin metabolism pathway after exposure to K56_Co_Che (Fig. 3B). As shown in Table 1, the RNA thermometer associated with KPHS_31750 (6-phosphofructokinase) displays regulatory characteristics as an attenuator (17). KPHS_31750 is also connected to the gene network (Fig. 3A), potentially contributing to the overall negative gene expression trend.

**TABLE 1:** Genomic RNA thermometer activity^*a*^

#ID	Genome	Strand	Associated_CDS	Start_genome	End_genome	Rfam_ID	Rfam_name	E_value	Score	Start_align	End_align
RNA_thermometer_1602	NC_016845.1	+	KPHS_31750	3159385	3159509	RF01859	Phe_leader	4.1E−16	74.1	1	125
RNA_thermometer_1660	NC_016845.1	+	KPHS_33570	3335790	3336228	RF01766	cspA	1.7E−41	145.6	1	439
RNA_thermometer_37	NC_016845.1	+	KPHS_00820	81970	82028	RF01859	Phe_leader	3.1E−05	23.6	1	37
RNA_thermometer_39	NC_016845.1	+	KPHS_00860	90457	90536	RF01859	Phe_leader	5.6E−06	27.6	1	80
RNA_thermometer_606	NC_016845.1	+	KPHS_11580	1215650	1215709	RF01859	Phe_leader	4.8E−06	27.3	1	60
RNA_thermometer_1537	NC_016845.1	+	KPHS_30060	2996291	2996377	RF01859	Phe_leader	0.00021	20.7	1	36
RNA_thermometer_1593	NC_016845.1	+	KPHS_31510	3134900	3134984	RF01859	Phe_leader	4.9E−05	23.4	1	85
RNA_thermometer_19	NC_016846.1	-	KPHS_p200210	7230	7296	RF01804	Lambda_thermo	0.00067	14.5	1	67

^*a*^ Three different RNA thermometers with eight associated genes were identified in the K56_Co Che condition. According to the initial ANNOgesic prediction, KPHS_31750 contains the associated coding sequence of a phenylalanine leader peptide RNA thermometer.

### Regulation of cobalamin in porphyrin metabolism

**Fig 4:**
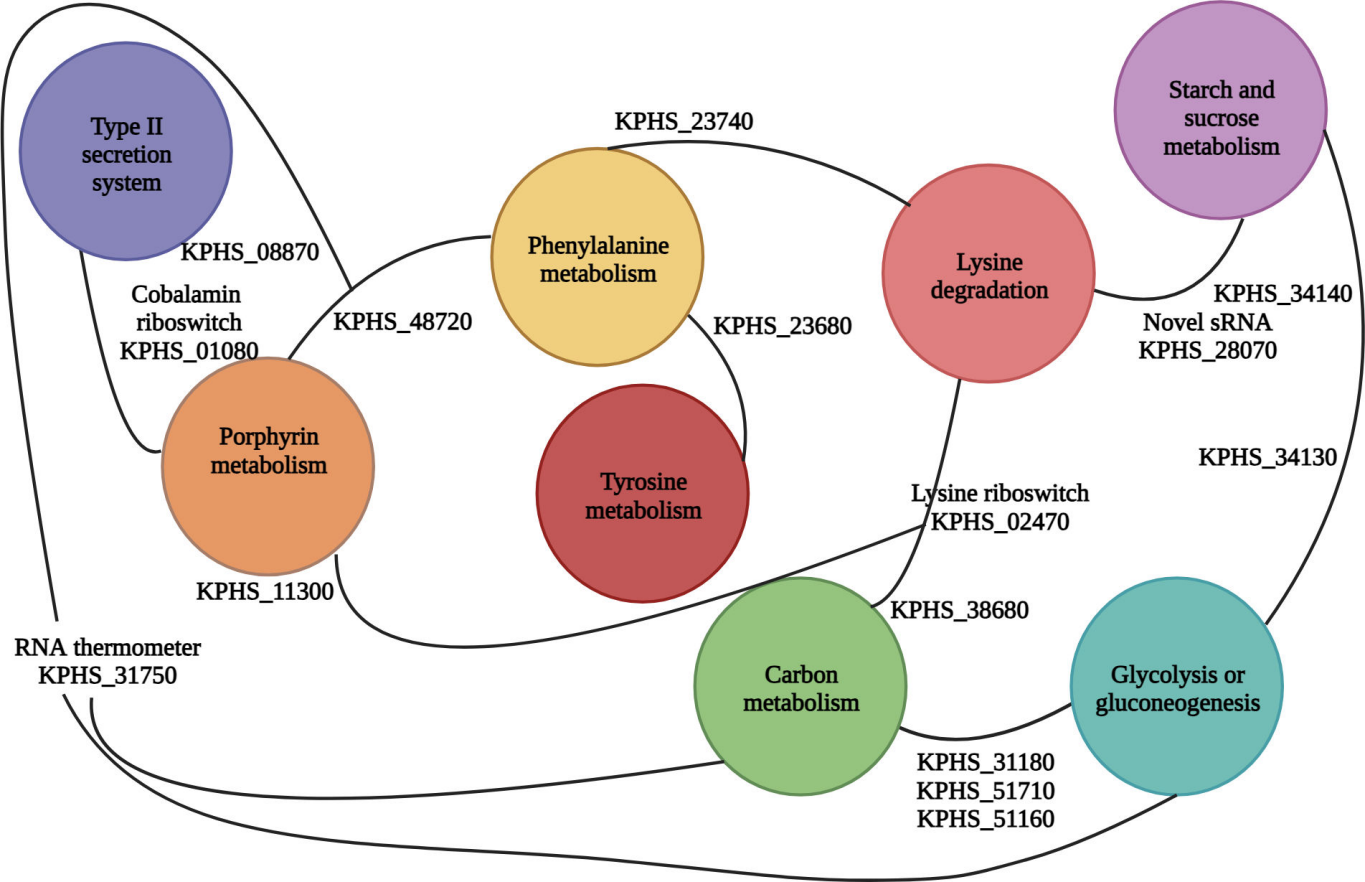
Pathway network analysis. Nodes represent specific pathways, and lines represent connections. Two functionally related genes, KPHS_23690 and KPHS_23710, are linked to the downregulation of KPHS_31750 (6-phosphofructokinase) through the downregulation of KPHS_48720 (bacterioferritin) in the porphyrin metabolism pathway. Additionally, KPHS_01080 is functionally linked to other genes involved in porphyrin metabolism and forms a link with KPHS_08870 (pullulanase D protein) in the type II secretion system. However, the downregulation of KPHS_11300 is associated with the upregulation of KPHS_02470, which is related to the lysine riboswitch. Furthermore, a novel sRNA targets KPHS_28070 and forms a network with KPHS_34140. KPHS_31180, KPHS_51710, and KPHS_51160 are upregulated in carbon metabolism, glycolysis, and gluconeogenesis.

**Fig 5:**
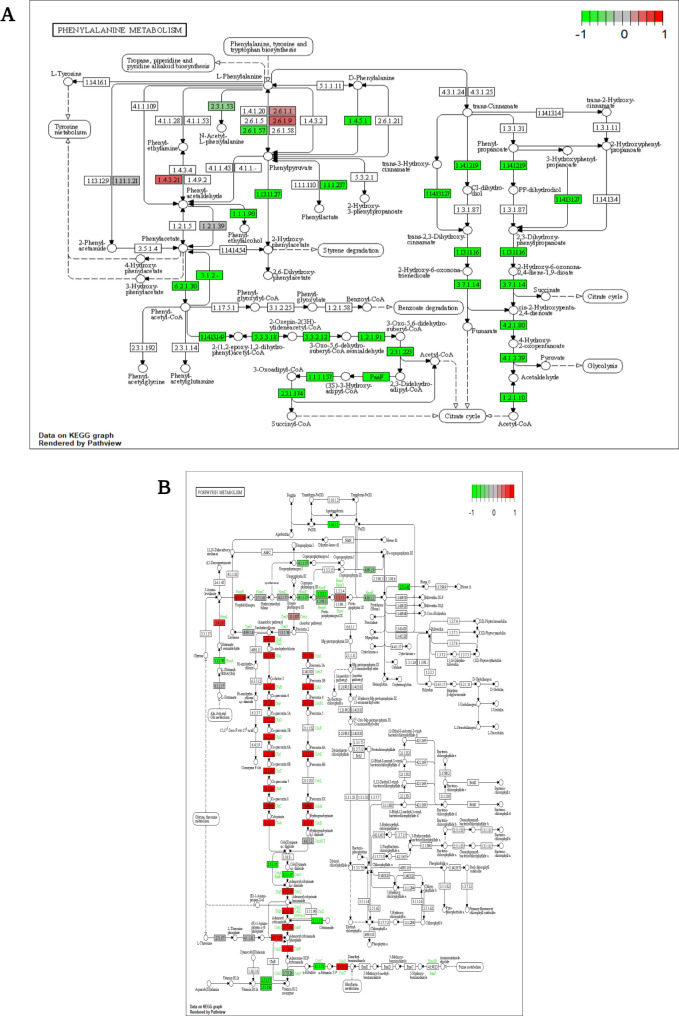
Kyoto Encyclopedia of Genes and Genomes (KEGG) pathways of metabolism. (**A**) Phenylalanine metabolism. Eventually, fewer intermediates were produced, such as acetyl-CoA and succinyl-CoA, from the citrate cycle according to the KEGG pathway analysis. (**B**) Porphyrin metabolism. The *de novo* pathway (anaerobic and aerobic pathway) is positively expressed, while the salvage pathway (transporter pathway) is negatively expressed.

Determination of the involvement of regulatory noncoding RNAs is essential to explore the interactions among pathways, even though the individual metabolic pathways have been described in the graphical representations (Fig. 4; Fig. S5). Notably, some of these connections represent regulatory RNAs. For instance, the absence of noncoding RNAs disrupts the circuit, indicating their important role. The ANNOgesic tool predicted the presence of a cobalamin riboswitch located between positions 117353 and 117540 (Table 2). This riboswitch is positioned upstream of the TonB-dependent gene KPHS_01080. KPHS_01080 functions as a cobalamin transporter and serves as a receptor for bacteriocins and bacteriophages (18–20). This cis-regulatory riboswitch controls the expression of associated genes in a negative feedback loop (21). Based on this information, other genes involved in cobalamin biosynthesis indicate significant upregulation, except for the associated gene KPHS_01080 (Fig. 3B and 5B).

**TABLE 2:** Genomic riboswitch activity^*a*^

#ID	Genome	Strand	Associated_CDS	Start_genome	End_genome	Rfam_ID	Rfam_name	E_value	Score	Start_align	End_align
riboswitch_52	NC_016845.1	+	KPHS_01080	117353	117540	RF00174	Cobalamin	1.4E−27	94.5	1	188
riboswitch_137	NC_016845.1	+	KPHS_02470	286829	287064	RF00168	Lysine	2.5E−38	132.1	1	236
riboswitch_1439	NC_016845.1	+	KPHS_27520	2757256	2757357	RF00059	TPP	5.1E−16	69.5	1	102
riboswitch_1659	NC_016845.1	+	KPHS_33510	3332260	3332362	RF00080	yybP-ykoY	4.5E−13	44.3	1	103
riboswitch_1774	NC_016845.1	+	KPHS_36130	3630799	3630896	RF00059	TPP	7.8E−16	68.6	1	98
riboswitch_2046	NC_016845.1	+	KPHS_42660	4297879	4298057	RF00174	Cobalamin	2.9E−29	100.1	1	179
riboswitch_2184	NC_016845.1	+	KPHS_45820	4607336	4607487	RF00050	FMN	2.5E−33	125.2	1	152
riboswitch_10	NC_016845.1	+	KPHS_00290	35097	35170	RF00519	suhB	5.6E−05	21	1	74
riboswitch_39	NC_016845.1	+	KPHS_00860	90473	90552	RF00516	ylbH	3.7E−05	21.1	1	80
riboswitch_1426	NC_016845.1	+	KPHS_27170	2724825	2724923	RF00442	ykkC-yxkD	0.00049	16.4	1	99
riboswitch_1610	NC_016845.1	+	KPHS_31920	3173316	3173390	RF00519	suhB	0.00017	19.1	1	75
riboswitch_105	NC_016846.1	+	KPHS_p201070	74176	74242	RF01051	c-di-GMP-I	0.00022	19.8	1	67

^*a*^ ANNOgesic analysis of the colistin and chemical #3 combination treatment predicted 10 riboswitches with 12 associated genes. Notably, KPHS_01080 was associated with the cobalamin riboswitch, and KPHS_02470 was associated with the lysine riboswitch.

### Enhancement of nutrient acquisition through the type II secretion system

The type II secretion system (T2SS) plays a crucial role in nutrient acquisition from the extracellular environment by releasing substrates that act as local and long-range effectors (22). It is a multi-protein complex that helps the transport of extracellular factors from the periplasm to the cell surface (23), and several T2SS components were upregulated (Fig. 2C; Fig. S6). KPHS_08870 (pullulanase D protein) relies on a sec transporter for its function (24) and utilizes a signal sequence-dependent pathway and a species-specific accessory mechanism to export proteins across cell membranes (25–27). KPHS_08820 (pullulanase I protein) is involved in the secretion of proteins as part of the pseudopilin complex (28). Both KPHS_08860 (pullulanase E protein) and KPHS_08790 (pullulanase L protein) may work in the opening and closing of the secretion pore (29) as an ATPase (30) and inner membrane element, respectively, to secrete toxins and hydrolytic enzymes (31).

### Similarities in the tyrosine metabolism and phenylalanine pathways

Another metabolic pathway that interacts with phenylalanine is tyrosine metabolism. Both pathways show an overall downregulated pattern, indicating a close relationship between aromatic amino acids. KPHS_06330 is involved in the degradation of 4-hydroxyphenyl acetic acid (32), and is functionally related to KPHS_23680 (phenylacetic acid degradation protein PaaN) (33) under combination treatment (Fig. 3D).

### Involvement of RNA thermometers in regulatory processes

The phenylalanine leader peptide attenuator is linked to KPHS_31750, which acts as a 6-phosphofructokinase in the carbohydrate metabolic process, specifically in the carbon metabolism and glucose or gluconeogenesis pathways. According to the Database for Annotation, Visualization, and Integrated Discovery (DAVID), KPHS_31180 (pyruvate kinase), KPHS_49540 (gluconate kinase), and KPHS_31750 (6-phosphofructokinase) are functionally related (34, 35). KPHS_31180 is associated with pyruvate conversion during glycolysis (36), and KPHS_51160 is involved in the glucose catabolic process. Upregulation of KPHS_49540 (gluconate kinase) is involved in the carbohydrate metabolic process, which includes thermoresistant and thermosensitive isozymes (37). KPHS_49540 catalyzes the phosphoryl transfer to produce gluconate-6-phosphate, a precursor of gluconate metabolism (38, 39). Consequently, an RNA thermometer regulates carbon metabolism, glycolysis, and gluconeogenesis (Fig. 3E). Furthermore, KPHS_51710 (triosephosphate isomerase) converts dihydroxyacetone phosphate to glyceraldehyde-3-phosphate dehydrogenase (GAPDH) (40), contributing significantly to energy production for survival. In the present study, KPHS_31520 (phosphoenolpyruvate synthase) was revealed to be responsible for phosphorylation. Fig. S7 shows that the RNA thermometer-associated gene KPHS_31750 and the lysine riboswitch-associated gene KPHS_02470 are indirectly linked through KPHS_21050 (glutamate dehydrogenase). Although no significant changes were observed in the expression of the KPHS_21050 gene itself, the gene may functionally interact with these regulators. Glutamate and phenylalanine exhibit structural and functional similarities due to the presence of conserved lysine residues that are important for catalytic activity (41). However, unlike KPHS_31750, a functional network between KPHS_33570 (cold shock protein) and significantly expressed genes was not observed in the KlebNet results. This finding contradicts the results of the ANNOgesic analysis (Table 1). One possible explanation is that our ANNOgesic analysis was not based on differential RNA sequencing (dRNA-seq) or term-seq. RNA-seq-based ANNOgesic analysis has limitations, such as the inability to predict bacterial transcription start sites, processing sites, and promoters. Therefore, further investigation is needed to clarify this discrepancy.

### Interconnection between carbon metabolism and glycolysis/gluconeogenesis

Carbon metabolism is closely associated with glycolysis and gluconeogenesis. In the anaerobic glyoxylate bypass pathway, downregulated genes, including KPHS_02300 (isocitrate lyase), KPHS_02290 (malate synthase), and KPHS_23760 (3-hydroxybutyryl-CoA dehydrogenase), contribute to the production of glucose from fatty acids. Conversely, three upregulated genes, KPHS_31180 (pyruvate kinase), KPHS_51710 (triosephosphate isomerase), and KPHS_51160 (phosphoglyceromutase), are significantly expressed during carbon metabolism, and glycolysis/gluconeogenesis (Fig. 3E and F). Anaerobic energy production is likely increased through glycolysis or gluconeogenesis rather than the glyoxylate cycle.

### Gene expression in glycolysis and gluconeogenesis for energy acquisition and survival

Gluconeogenesis, the reverse process of glycolysis, is responsible for synthesizing glucose as a carbon source (42, 43). Reduced KPHS_03180 (acetyl-CoA synthetase) expression may result in decreased acetyl-CoA levels. In response to combination treatment, downregulation of aldehyde dehydrogenase leads to decreased production of acetate from ethanol (44). Reduced GAPDH (KPHS_26520) expression is associated with decreased bacterial pathogenesis (45). Additionally, upregulation of the glycoside hydrolase enzyme KPHS_36400 works in starch and sucrose metabolism (Fig. 2F). KPHS_29390 (6-phospho-beta-glucosidase), KPHS_43980 (6-phospho-beta-glucosidase), and KPHS_38950 (6-phospho-beta-glucosidase) participate in the cellulose catabolic and carbohydrate metabolic processes by hydrolyzing glycosidic bonds.

### Disruption of fatty acid membranes by lysine degradation

The presence of a functional network involving carbon metabolism and lysine degradation (Fig. 3G) indicates the involvement of lysine in the catalytic mechanism. The highly expressed protein KPHS_13090 (lysine decarboxylase 1) acts as an enzyme, catalyzing the decarboxylation of lysine for amino acid metabolism (46). Contrarily, the downregulation of KPHS_09840 (glutarate 2-hydroxylase/carbon starvation-induced protein) participates in the L-lysine degradation pathway by catalyzing glutamate hydroxylation to produce L-2-hydroxyglutarate (47). Another contributor to the pathway is the downregulated protein KPHS_43350 (acetyl-CoA acetyltransferase), which affects fatty acid degradation. Additionally, KPHS_23740 (enoyl-CoA hydratase-isomerase) is significantly downregulated in both phenylalanine metabolism and lysine degradation. It represents catalytic activity as an enoyl-CoA delta isomerase for the degradation of unsaturated fatty acids (48). This enzyme also plays a role in membrane disruption by altering phospholipid membranes and fatty acid synthesis (49).

**Fig 6:**
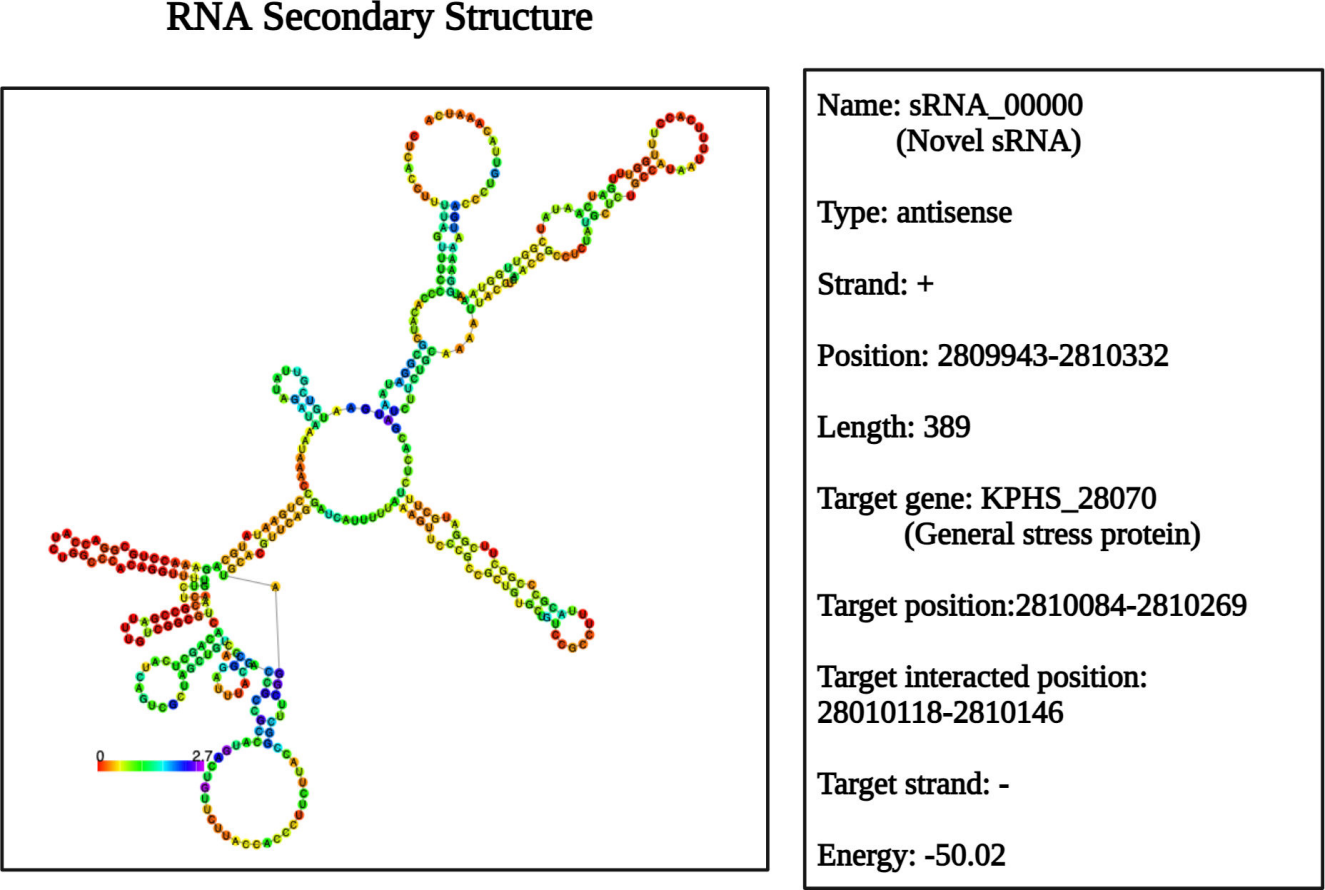
Identification of a novel sRNA by ANNOgesic. The sRNA is an antisense RNA with a length of 389 nucleotides that regulates gene expression via negative binding energy. This cis-acting antisense sRNA is transcribed in the opposite strand of the target gene, KPHS_28070. This gene is a general stress protein, related to the stress-induced acidophilic repeat motif-containing protein.

Nevertheless, transmission electron microscopy results did not reveal significant morphological changes in the structure (Fig. S8), presumably due to a twofold decrease from the minimum inhibitory concentration (MIC). According to the ANNOgesic analysis, a novel sRNA can target the KPHS_28070 (stress-induced bacterial acidophils repeat motif) gene (Fig. 6). This negatively expressed gene is related to acid stress and can cause bacteriostasis (50). The lysine riboswitch-associated KPHS_02470 (glucose-6-phosphate isomerase) gene was indirectly linked to the functionally associated KPHS_13980 (carboxylate-amine ligase) gene (Fig. 3G). Within the lysine degradation pathway, this gene may function under lysine riboswitch regulation.

### Impact of starch and sucrose metabolism on bacterial durability

In the last starch and sucrose metabolism pathway (Fig. 2H), KPHS_34140 (trehalose-6-phosphate phosphatase) downregulation controls the dephosphorylation of trehalose-6-phosphate to produce trehalose and orthophosphate under stressful conditions (51). Oppositely, the upregulation of KPHS_04990 (trehalose(maltose)-specific PTS system component IIBC) is associated with the degradation of trehalose-6-phosphate to glucose and glucose 6-phosphate in the carbohydrate transport system. To achieve a maximum growth rate, bacteria reduce carbon source utilization and increase glucose production (52). Increased KPHS_49390 (glycogen phosphorylase) expression is related to the formation of glycosidic bonds. Inversely, the downregulation of KPHS_28030 (putative glycosidase) expression plays a role in hydrolyzing the glycosidic bond and is involved in carbohydrate metabolism. The overall trend indicates an increase in glycosidic bond formation. The highly upregulated KPHS_04980 (trehalose 6-P hydrolase) helps protect against hyperosmotic and thermal stress (53).

KPHS_52250 (putative PTS, EIIC), part of the phosphoenolpyruvate-dependent sugar PTS, induces metabolism and transcription (54). This system regulates sugar transport and the phosphorylation process (55). Its downregulation negatively affects sugar uptake. The upregulation of KPHS_04990 also works in a similar area. KPHS_52250, along with downregulation of KPHS_50400 (cellulose synthase catalytic subunit), inhibits starch saccharification and nutrient acquisition (56). KPHS_52250 also interacts with upregulated glycoside hydrolase genes, such as KPHS_29390, KPHS_43980, and KPHS_38950, from the glucose or gluconeogenesis pathway (Fig. 2F and H).

## DISCUSSION

Gram-negative bacteria have a wavy double-layered membrane structure with a thin peptidoglycan layer (57), and some lipopolysaccharides produced by these bacteria can act as endotoxins. Therefore, invasion of the cell envelope of Gram-negative bacteria is a necessary step for infection. The physiochemical property of chemical #3 in this study may lead to strong interaction with colistin. The counteractive interplay between cell surface stress and metabolic rewiring plays a vital role in the balance between the effect of the combination treatment and bacterial homeostasis. The present study provides evidence supporting the perspective that lysine degradation, starch and sucrose metabolism, glycolysis, and gluconeogenesis contribute to the vulnerability of bacterial surface protection against the combined effects of colistin and chemical #3. Carbohydrates are important for bacterial energy storage and cell envelope integrity (58), and are a substantial component of peptidoglycans. Reduced carbohydrate biosynthesis affects glycosyltransferase activity and glycosidic bond formation. Furthermore, altered fatty acid composition leads to changes in phospholipid membrane properties, and phenylalanine and tyrosine metabolism contribute to energy deficiency.

Under antimicrobial pressure, carbon metabolism, as well as starch and sucrose metabolism, participate in energy production as part of the survival strategy in strain K56. Bacterial infections involve the secretion of proteins into the extracellular environment via the T2SS as a virulence mechanism (59). It is worth noting that cobalt ions promote pullulanase activity during T2SS secretion (60). Porphyrin metabolism also enhances cobalamin biosynthesis, which serves as a cofactor regulating gene expression (61). KPHS_42460 is involved in the intermediate step of decreasing biosynthesis of the cobalamin ligand (62). Both KPHS_42480 and KPHS_42640 catalyze amidation during cobalamin biosynthesis (63) and show enhanced expression under aerobic conditions in the late step of cobalt insertion and under anaerobic conditions in the first step of cobalt insertion in the cobalamin biosynthetic process (64). Decreased KPHS_11300 expression leads to a reduction in the heme biosynthetic process. Throughout this process, regulatory RNA elements modulate the expression of functionally related genes, forming a systemic network that fine-tunes gene expression for regulation.

The RNA thermometer, specifically the phenylalanine leader peptide, is implicated in nutritional and energy control, although additional investigation is required to establish its precise role. This structure relies on temperature or starvation conditions to modulate gene expression (65) by adjusting the secondary structure and affecting ribosome binding site exposure (66), which in turn influences translation in response to heat shock or cold shock.

Another strategy involves exploring the functionality of novel sRNAs and their interaction with the target gene KPHS_28070 (Fig. 6). Antisense sRNAs can inhibit sRNA function by binding to sRNAs before they can interact with target mRNAs (67), which relieves translational repression and promotes gene expression (68). Utilization of novel sRNAs targeting KPHS_28070, a general stress protein, holds promise as a future therapeutic approach for regulating metabolism based on specific nucleotide sequences (69).

Riboswitches, regulatory segments of mRNA, play a critical role in controlling gene expression by binding to small molecules at their aptamer domains. This binding can switch gene expression on or off depending on the presence or absence of the ligand. The lysine riboswitch, for example, regulates lysine biosynthesis by inversely modulating citrate synthase activity in the tricarboxylic acid cycle (70). Targeting the lysine riboswitch could be an effective approach for remotely controlling bacterial cell growth. Similarly, the cobalamin riboswitch holds promise for various applications due to its specificity to bacteria and safe host interaction. Association with KPHS_01080 (cobalamin transporter) is crucial for utilizing the riboswitch as a delivery system for treatments.

The current study highlights the potential of combination treatment with colistin and chemical #3 as a starting point for addressing multidrug-resistant *K. pneumoniae*. Through a comprehensive analysis, this study elucidates important directions by unraveling the intricate functional network between the mode of action of the novel combination treatment and the bacterial survival mechanisms. This systematic interpretation will allow the identification of specific pathways and noncoding RNAs that play significant roles in multidrug resistance. However, further biotechnology, pharmacology, and synthetic biology studies are required to validate the effectiveness of the identified candidates. Nonetheless, this study serves as a fundamental basis for future investigations in drug discovery and therapeutic approaches aimed at combating antibiotic resistance.

## MATERIALS AND METHODS

### Chemical library screening and compounds

To evaluate the effect of chemical compounds on K56, the strain was exposed to sub-MIC meropenem (8 µg/mL) and a chemical compound library (6,696 compounds) was obtained from the Korea Chemical Bank. Synergistic screening was conducted using the broth microdilution technique, following the guidelines of the Clinical and Laboratory Standards Institute. Additionally, 1,142 similar compounds from the same source were included in the screening. The experimental procedure was identical for all compounds (Fig. S8 and S9).

### Checkerboard studies

A single colony of the K56 strain was selected and adjusted to a 0.5 McFarland standard. The colony was further diluted (1:100) in cation-adjusted Mueller–Hinton Broth before inoculation. Serial dilutions of meropenem (Yuhan, Seoul, South Korea), colistin (Sigma-Aldrich, St. Louis, MO, USA), chemical #3 (LegoChem Biosciences, Daejeon, South Korea), and triclosan (Sigma-Aldrich) were prepared. The MIC was determined as the lowest concentration with no visible growth. The fractional inhibitory concentration index (FICI) was calculated using the following formula: FICI = FICA + FICB = MICAB/MICA + MICBA/MICB, where MICAB represents the MIC of compound A in combination with compound B, MICA represents the concentration of compound A alone, MICBA represents the concentration of compound B with compound A, and MICB represents the MIC of compound B alone (Fig. S10). The FICI interpretation range was as follows: FICI ≤ 0.5 indicated synergy, 0.5 < FICI ≤ 1 indicated an additive effect, 1 < FICI ≤ 2 indicated no interaction, and FICI > 2 indicated antagonism (Table S3).

### Bacterial growth curve evaluation

A 96-well clear, flat-bottomed microplate (SPL Life Sciences, Kyeonggido, South Korea) and optical adhesive films (Thermo Fisher Scientific, MA, USA) were used for growth curve measurements in a microplate reader (Molecular Devices, CA, USA). The microplate reader was set to measure the optical density at 600 nm every 10 minutes for 24 hours. The midpoint of the logarithmic phase of bacterial growth was determined. Error bars represent the standard deviation. Statistical analysis was performed using student’s *t*-test with GraphPad Prism v9.3.0 (Fig. S11).

### RNA extraction

The treatment conditions included 0.5 µg/mL colistin and 1 µg/mL chemical #3 diluted with Luria broth in round-bottom tubes. After 14 hours and 50 minutes of incubation, the cells were harvested and subjected to RNA extraction using the RNeasy mini kit and RNase-free DNase set (Qiagen, Hilden, Germany) according to the manufacturer’s instructions. RNA quality was assessed using a NanoDrop spectrophotometer (Thermo Fisher Scientific).

### Transcriptome analysis

The Illumina total RNA-seq assay was performed using a stranded total RNA library prep kit. Library sequencing was carried out using the Nextseq550 sequencing platform at the Yonsei University Genome Center. The RNA-seq data were analyzed by the Bioinformatics Collaboration Unit at Yonsei University College of Medicine. In the processing and quality control steps, bcl format files were converted to fastq files using bcl2fastq2 (v2.20.0.422). Adapter and quality trimming were performed using Cutadapt (v1.18) (71) and Trimmomatic (v0.32) (72). Quality checks were conducted before and after trimming using FastQC (v0.11.7) (Table S4) (73). Alignment and transcriptome assembly were performed on the reference genome of *K. pneumoniae* HS strain 11286 using HISAT2 (v2.1.0) (74) and StringTie (v2.0.6) (75). Differential expression analysis was performed using the statistical algorithm LPEseq (76). Normalized count values, calculated *P*-values, and q-values were provided without replicates.

### Data processing and analyses

The READemption tool (v2.0.3) (77) was used to generate a coverage wiggle file from the fastq files of the total RNA-seq data. The ANNOgesic tool (v1.1.14) was utilized (11) to detect subcommand sRNA and its targets using RNAplex. Prediction of riboswitches and RNA thermometers was performed using the Rfam and Infernal databases (Sung-Huan Yu, 2018, www.github.com/Sung-Huan/ANNOgesic). Network-based functional analysis was conducted using KlebNet (3) and Cytoscape (v3.9.1) (78). Clustering heatmaps and pathway analysis visualization were performed using Perseus (79). The Venn diagrams in the supplementary figures were drawn with VENNY (2.1.0) (80).

## Data Availability

The data set (accession number GSE233610) will be publicly available from July 1, 2023.

## SUPPLEMENTAL MATERIAL

Supplemental material msystems.00877-23-s0001.docx Supplemental figures and tables.

Table S2 msystems.00877-23-s0002.xlsx Differentially expressed genes.

ASM does not own the copyrights to Supplemental Material that may be linked to, or accessed through, an article. The authors have granted ASM a non-exclusive, world-wide license to publish the Supplemental Material files. Please contact the corresponding author directly for reuse.
