# Supplementary material for: Use of a combined antibacterial synergy approach and the ANNOgesic tool to identify novel targets within the gene networks of multidrug-resistant Klebsiella pneumoniae
Source: mSystems. 2024 Feb 13;9(3):e00877-23. doi: 10.1128/msystems.00877-23 (PMC10949472; doi:10.1128/msystems.00877-23)
Supplement: Supplemental material — Supplemental figures and tables. [file msystems.00877-23-s0001.docx]

**ADDITIONAL FILES**

**Table S1.** K56 resistome data from ResFinder.

**
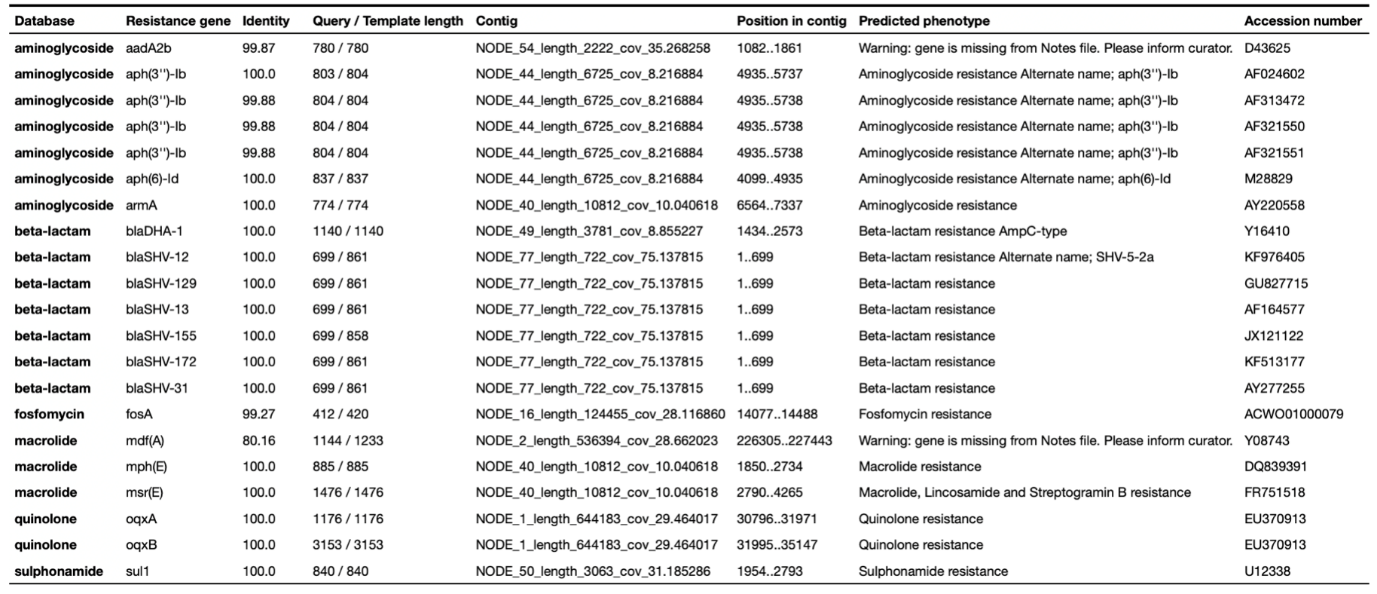
**

**Table S3.** Checkerboard assay to determine phenotypical susceptibility. A checkerboard assay was performed for phenotypic susceptibility determination. The combinations of 0.5 µg/ml colistin with 2 µg/ml chemical #3 and 1 µg/ml colistin with 1 µg/ml chemical #3 resulted in fractional inhibitory concentration index values much lower than 0.5, indicating a strong interaction. Chemical #3 and triclosan exhibited synergistic effects in the experimental results. However, caution is advised when considering chemical #3 as a potential therapeutic compound due to its similarity to triclosan, which has known negative effects and toxicity.


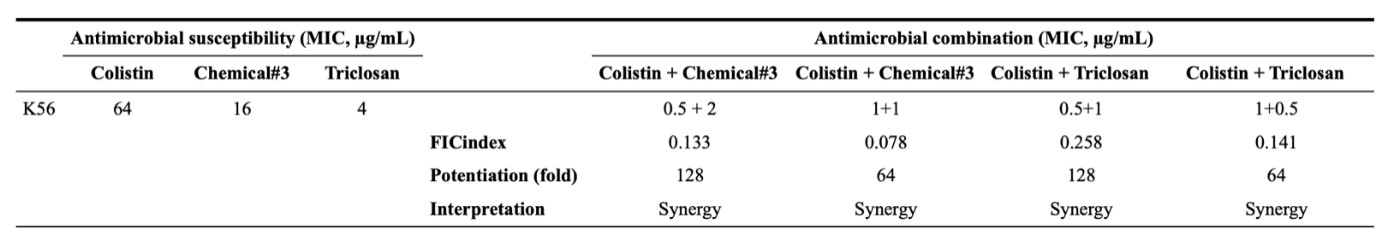


**Table S4.** Quality control reports from FastQC. From the alignment with HISAT2, the read depth and mapping rate were around 40M and ~90%, respectively. The READemption created coverage files of the wiggle format (77). For coverage calculation, different wiggle files were created with three different normalization methods (77). There was no normalization with raw counting values. The others were normalized by the total number of aligned reads and multiplied by the lowest number of aligned reads of all libraries, and normalized by the total number of aligned reads and multiplied by 1 million (78).


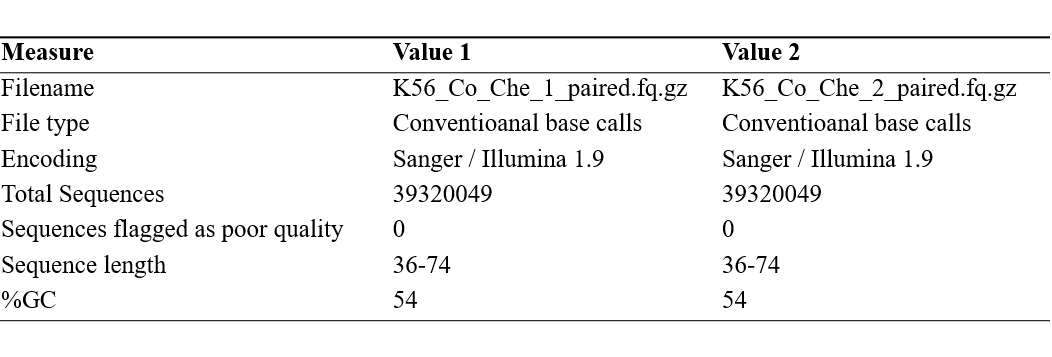


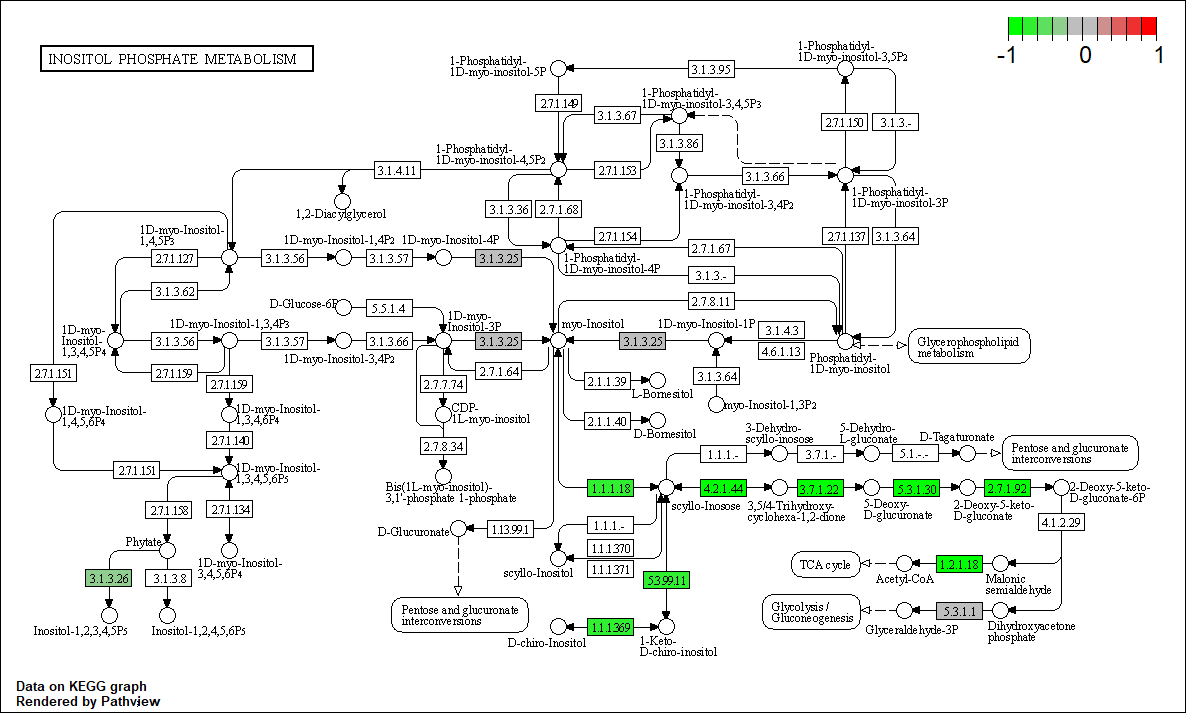


**Figure S1A.** Inositol phosphate metabolism of chemical #3 single treatment.
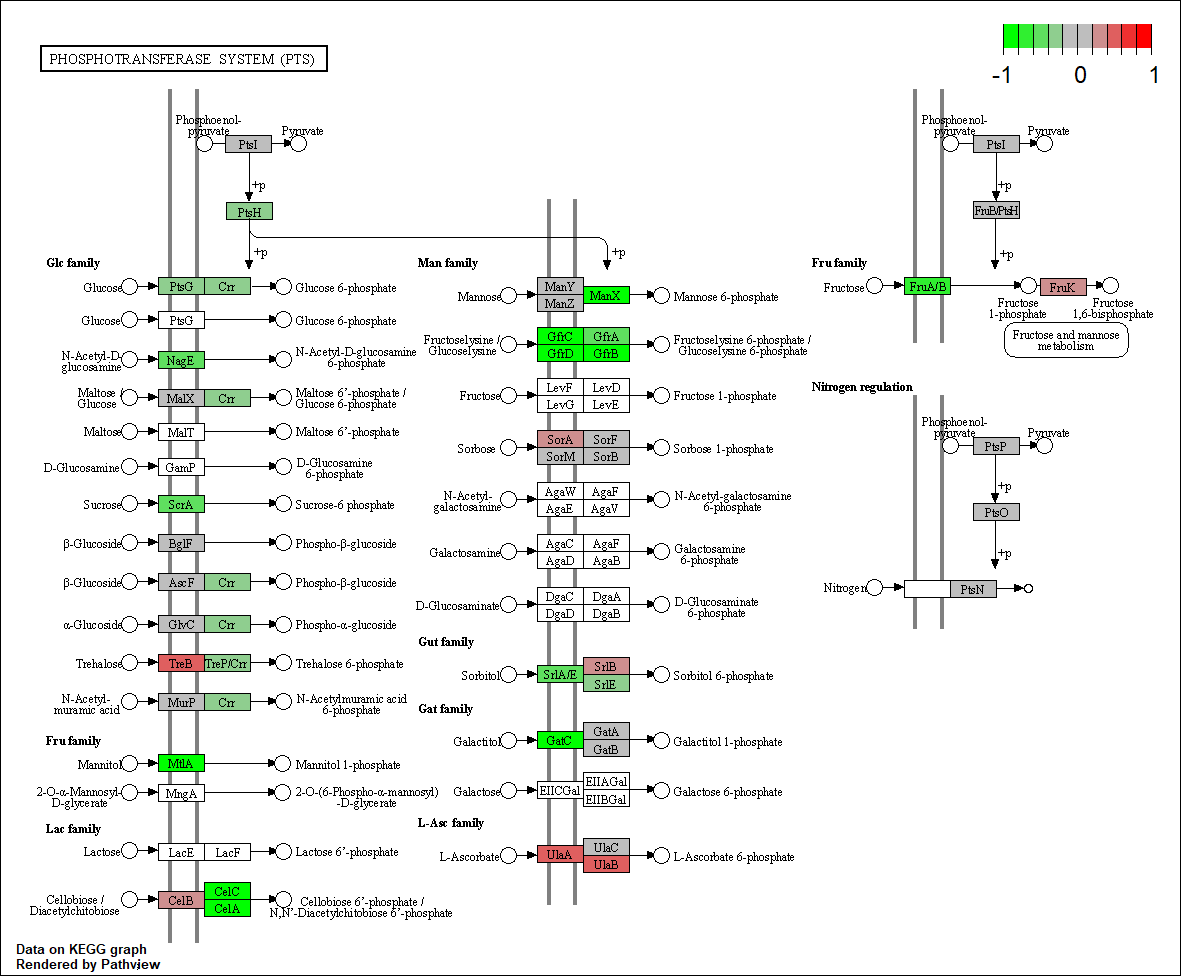


**Figure S1B.** Phosphotransferase system of chemical #3 single treatment.


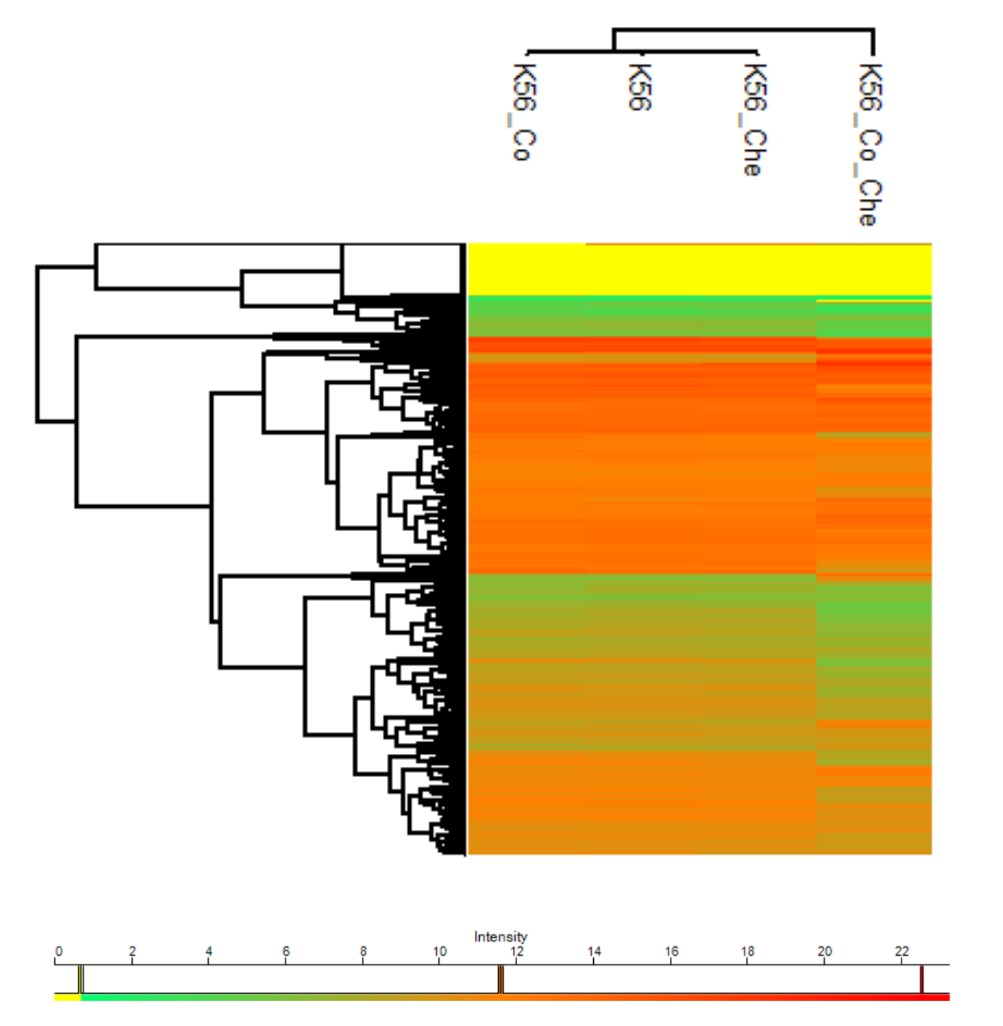


**Figure S2.** Hierarchical clustering and heatmap. Clustering analysis demonstrated that the control group (K56) and the two single-treatment groups exhibited similar patterns, but differences were observed for the combination treatment with colistin and chemical #3.


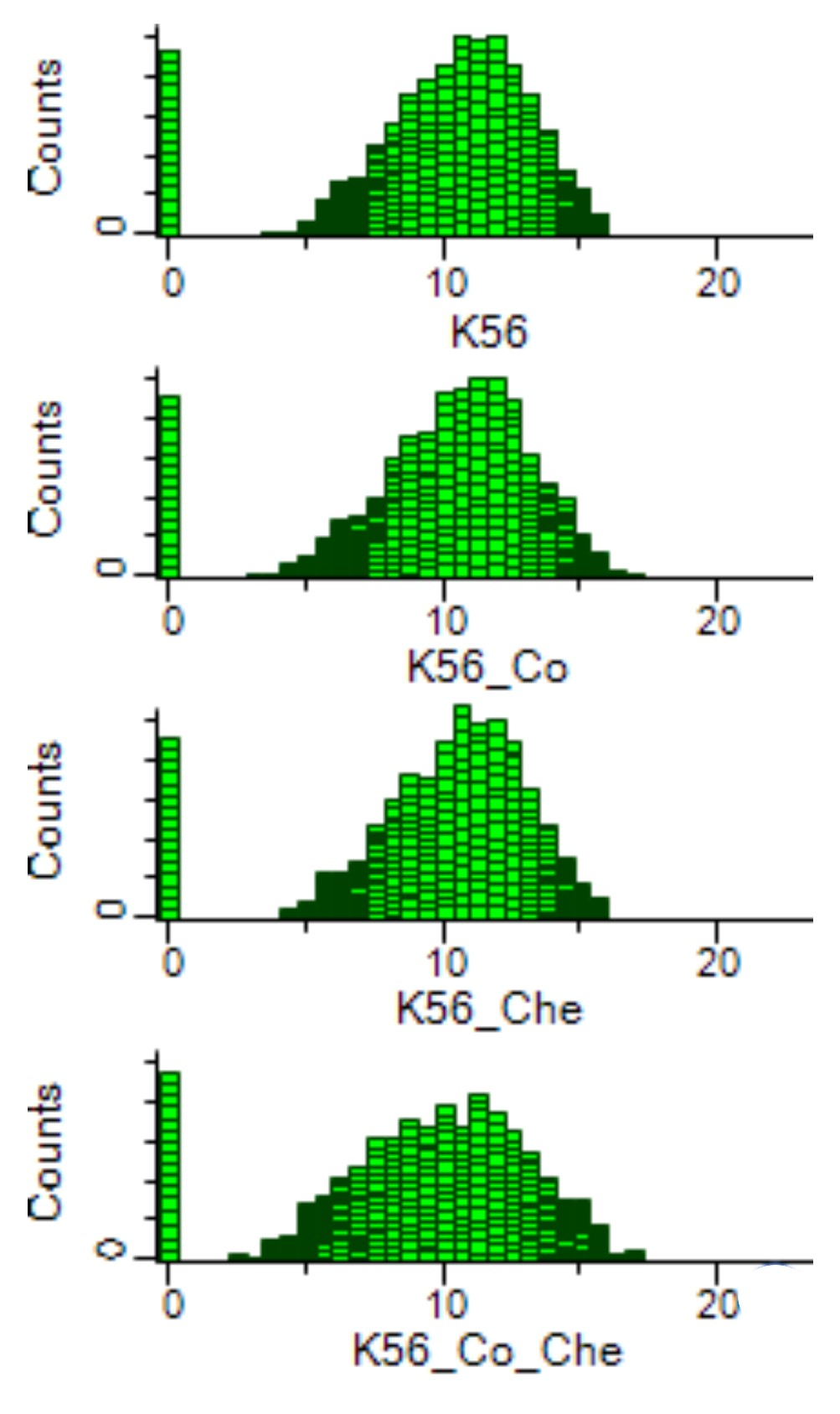


**Figure S3.** Histograms depicting the gene distribution. Histograms represent the gene counts vs. numerical values, and correspond to the interpretation of multiple scatter plots.


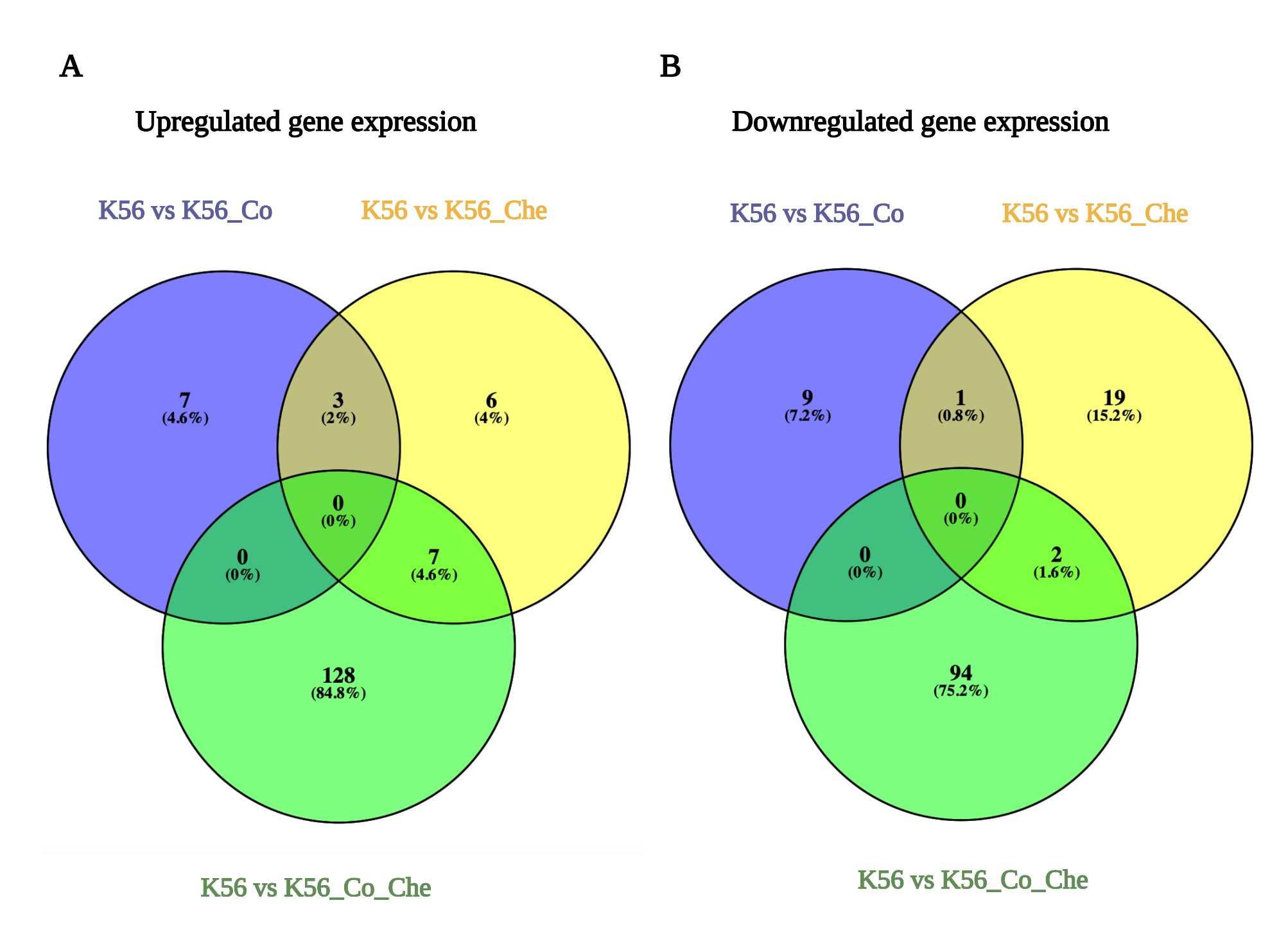


**Figure S4.** Venn diagrams. Panel (A) represents upregulated genes, and panel (B) represents downregulated genes. Purple, yellow, and green areas in panel (A) correspond to 7, 6, and 128 genes with increased expression, respectively. In panel (B), 9, 19, and 94, genes exhibited decreased expressions. The green exclusive areas in both panels were involved in various pathways related to the mode of action of the combination treatment. The central common area did not contain overlapping genes in terms of expression, indicating no shared properties among the three different treatments.


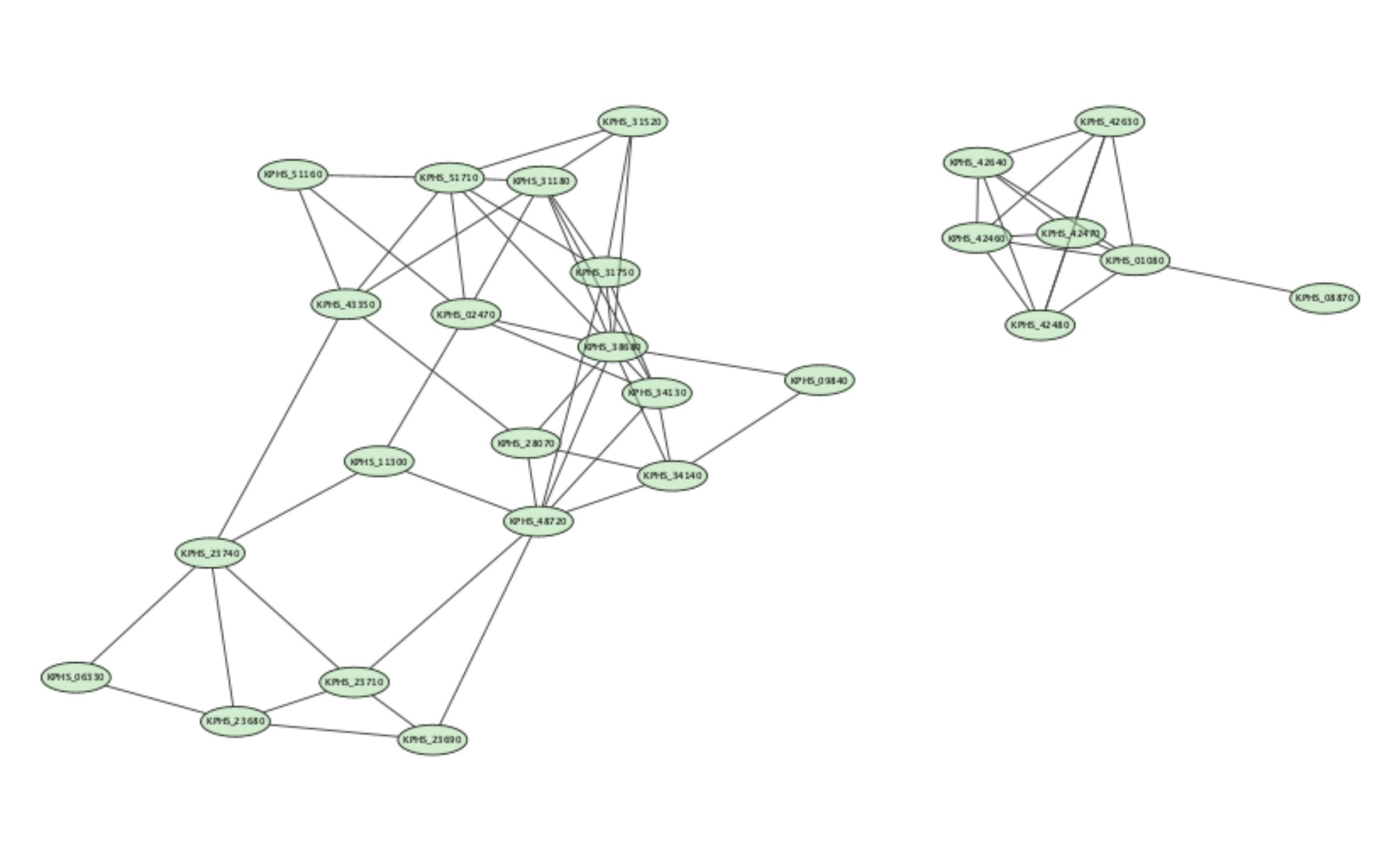


**Figure S5.** Integrated functional network.


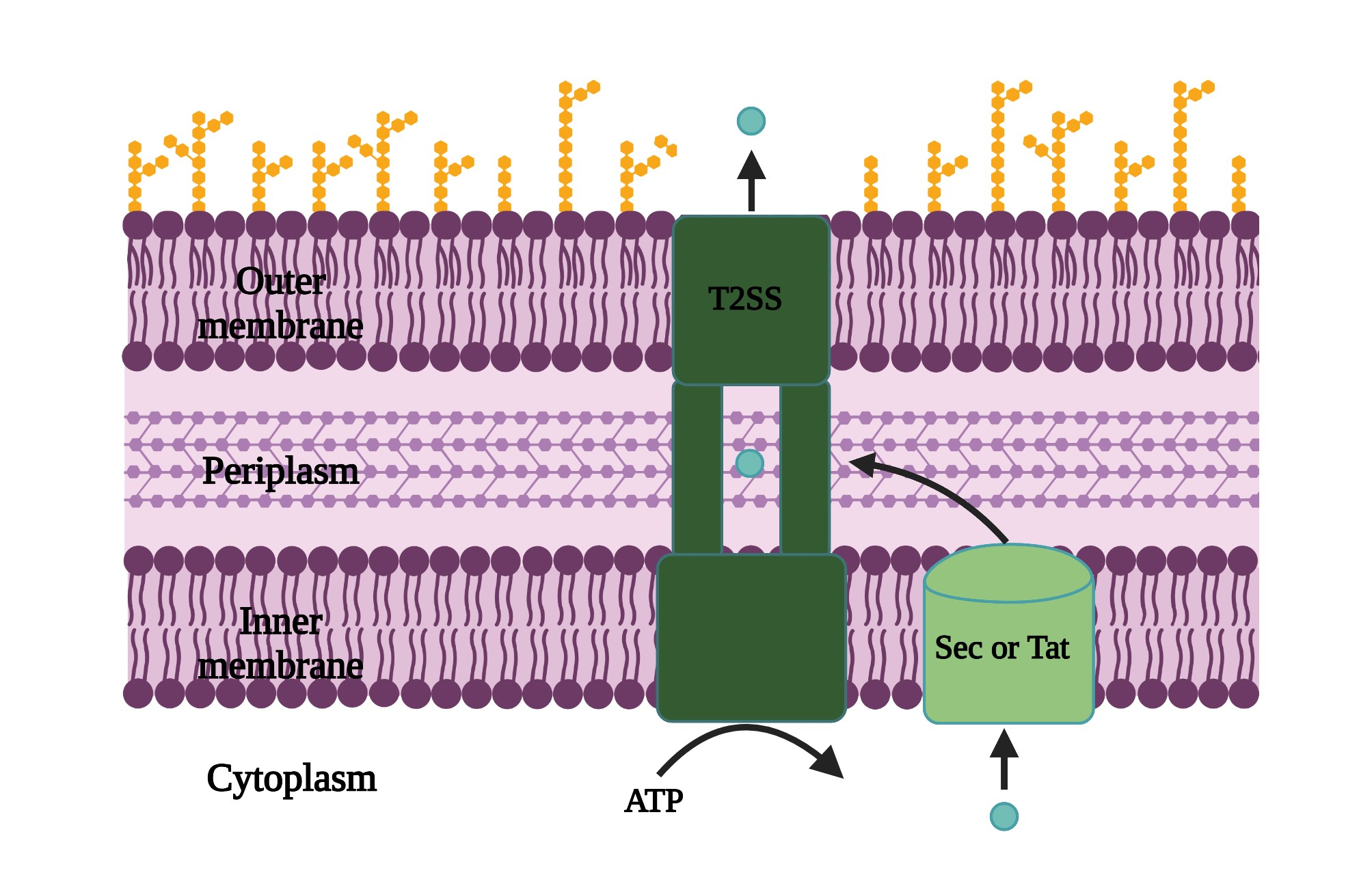


**Figure S6.** Mechanism of the type II secretion system.


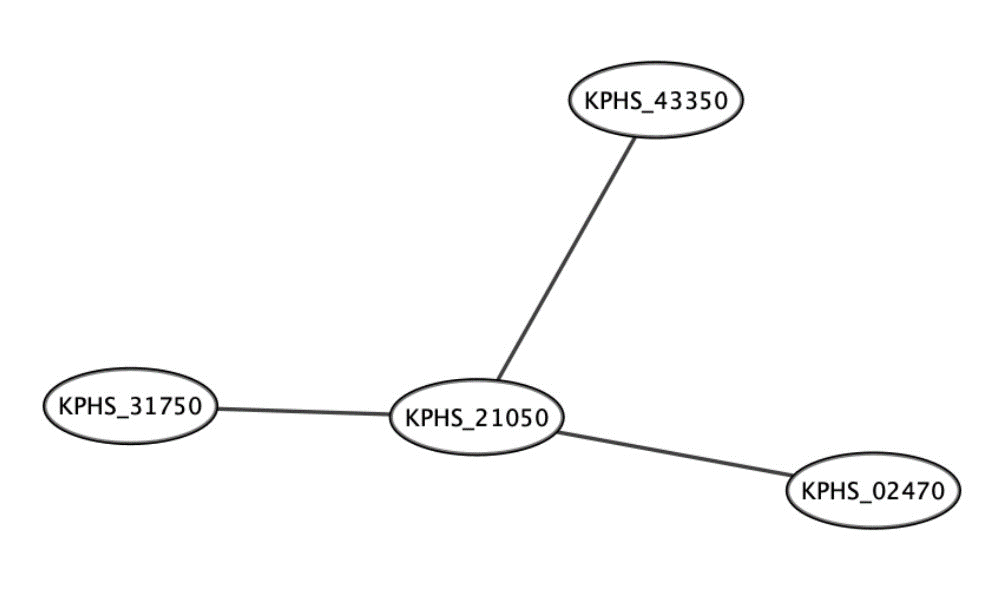


**Figure S7.** Genes associated with the phenylalanine leader peptide RNA thermometer.


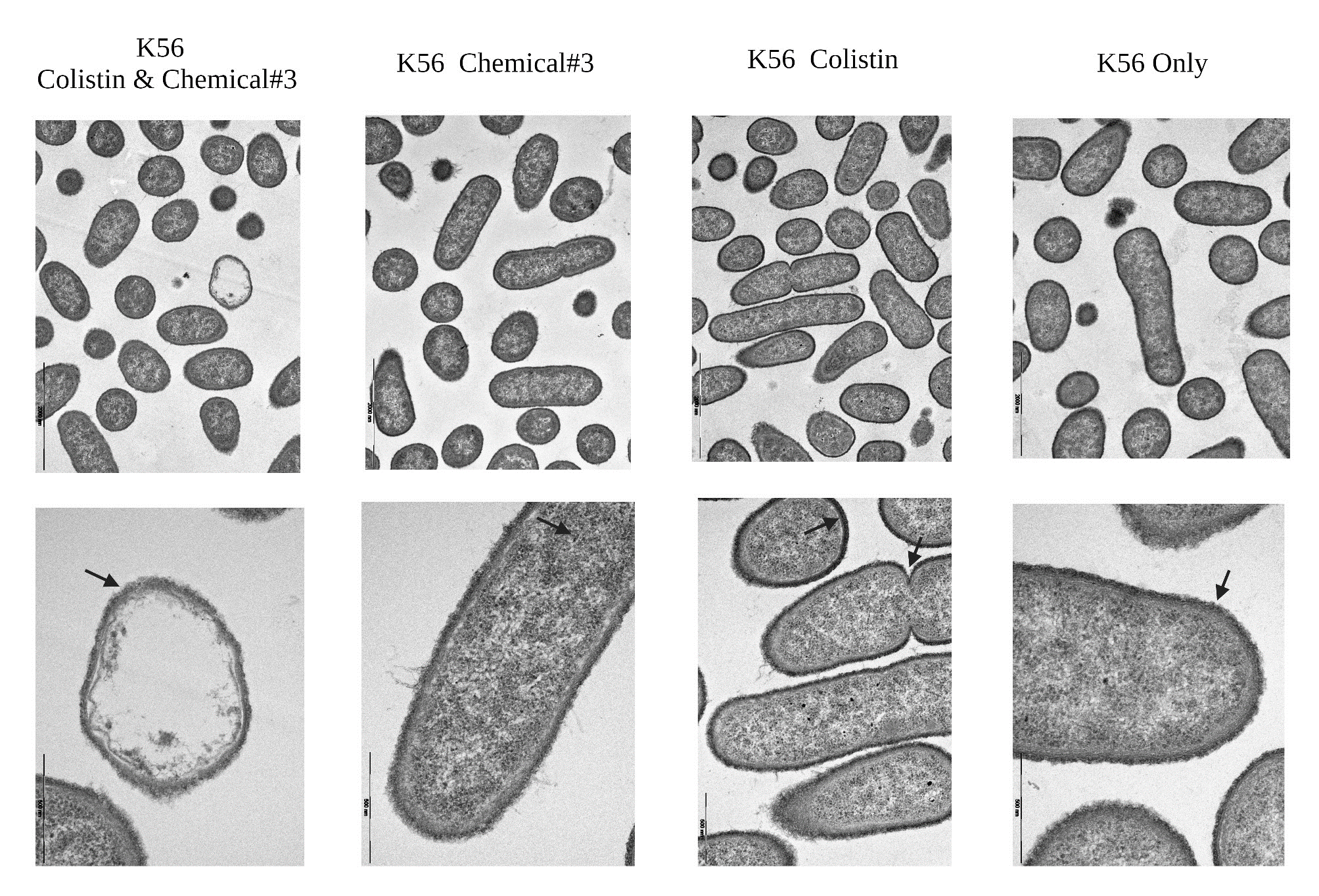


**Figure S8.** Transmission electron microscopy. No significant morphological differences were observed after treatment with 0.25 µg/ml colistin and 0.5 µg/ml chemical #3.


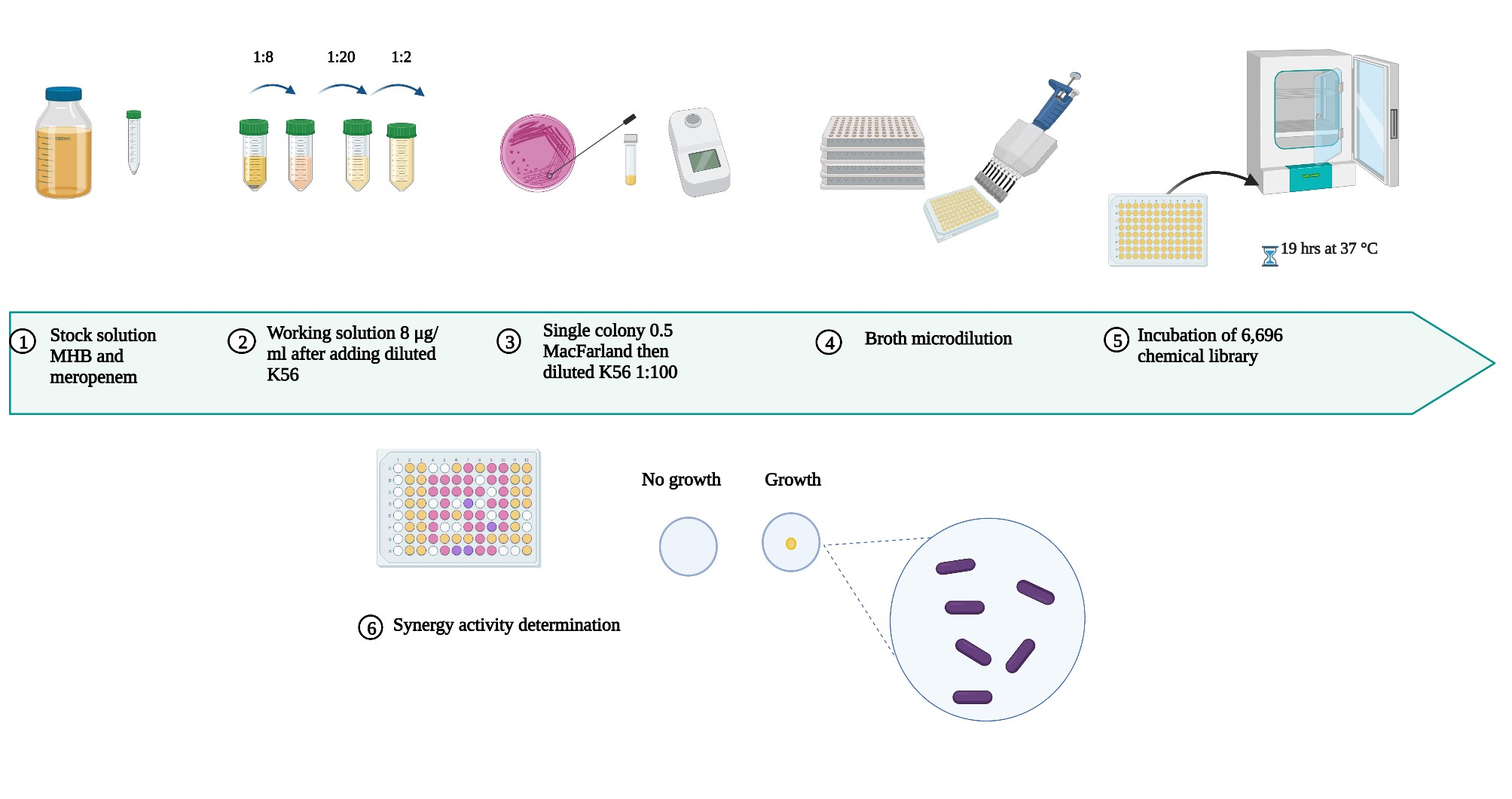


**Figure S9.** Broth microdilution technique for phenotypic characterization.


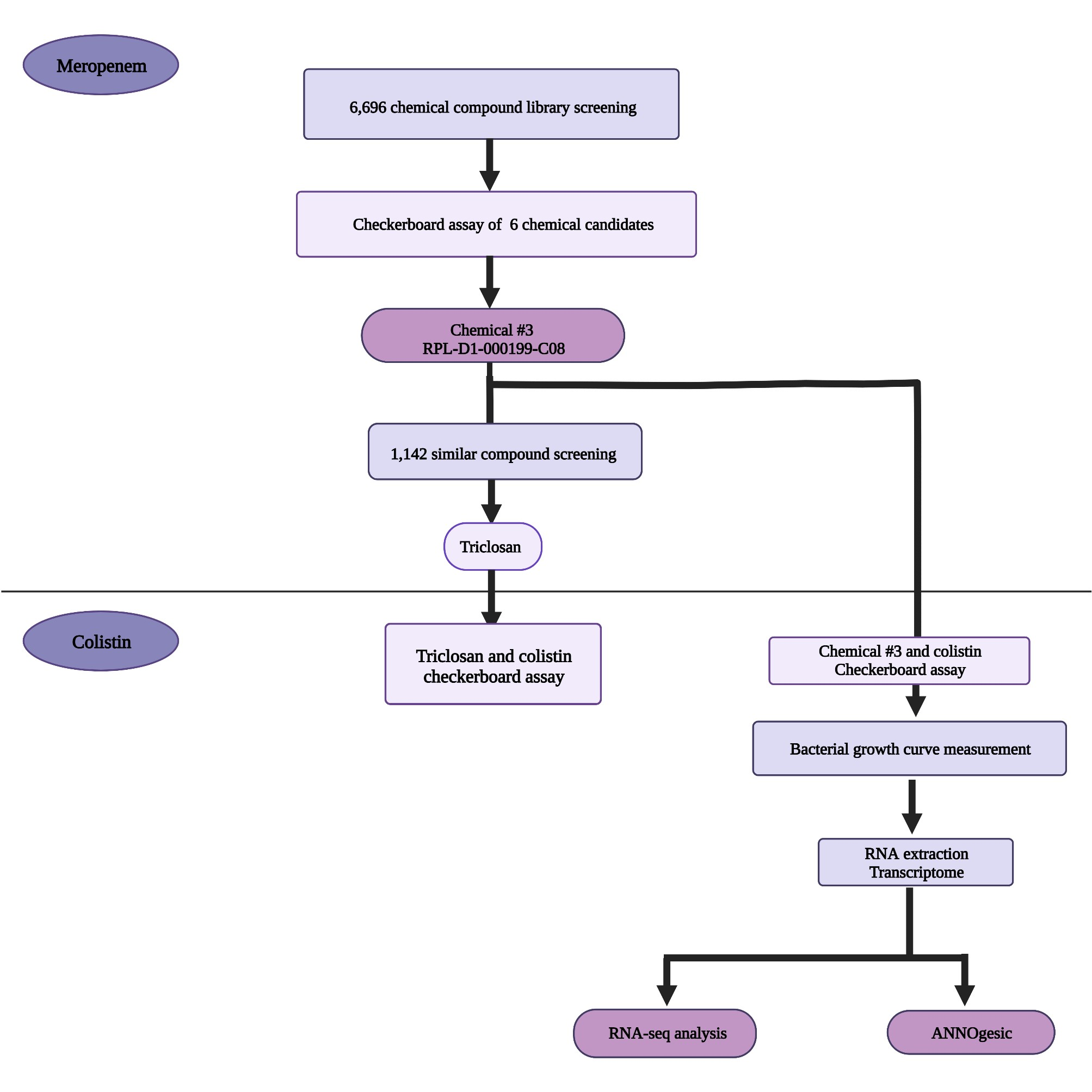


**Figure S10.** Overview of phenotypic and genotypic approaches.


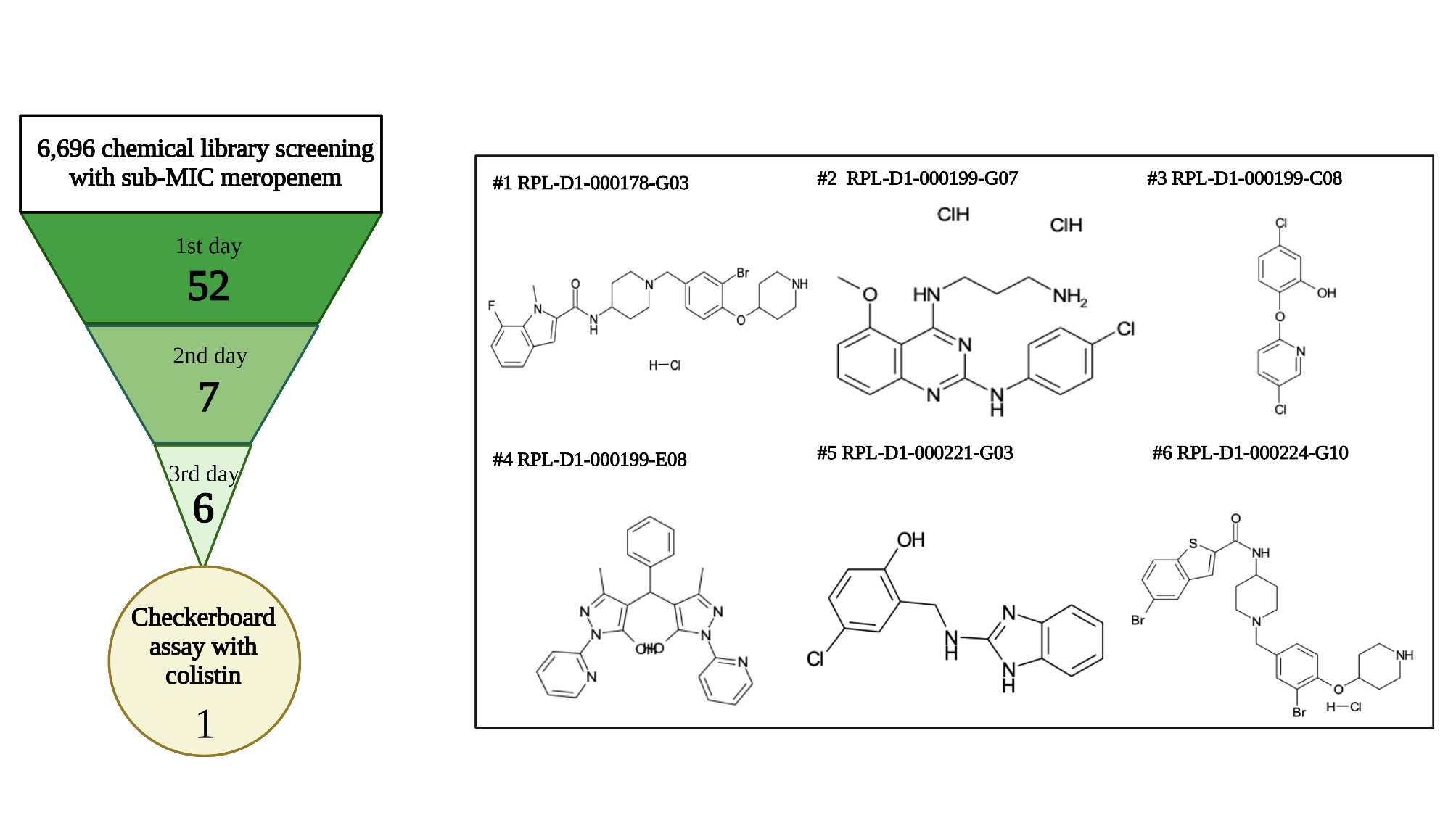


**Figure S11.** Screening of a library of 6,696 chemical compounds. Screening was conducted using a sub-MIC (8 µg/ml) concentration of meropenem to target the multidrug-resistant strain K56. After 3 days of observation, six candidates with synergistic activity were obtained. Checkerboard assay was then performed to determine the susceptibility of K56 to the six chemicals combined with meropenem. Only chemical #3 (RPL-D1-000199-C08) showed an additive effect when combined with meropenem. Chemical #3 was further tested with colistin, and strain K56 remained phenotypically susceptible. Additionally, 1,142 similar compounds were tested under the same conditions, revealing that triclosan exhibited synergy with meropenem.


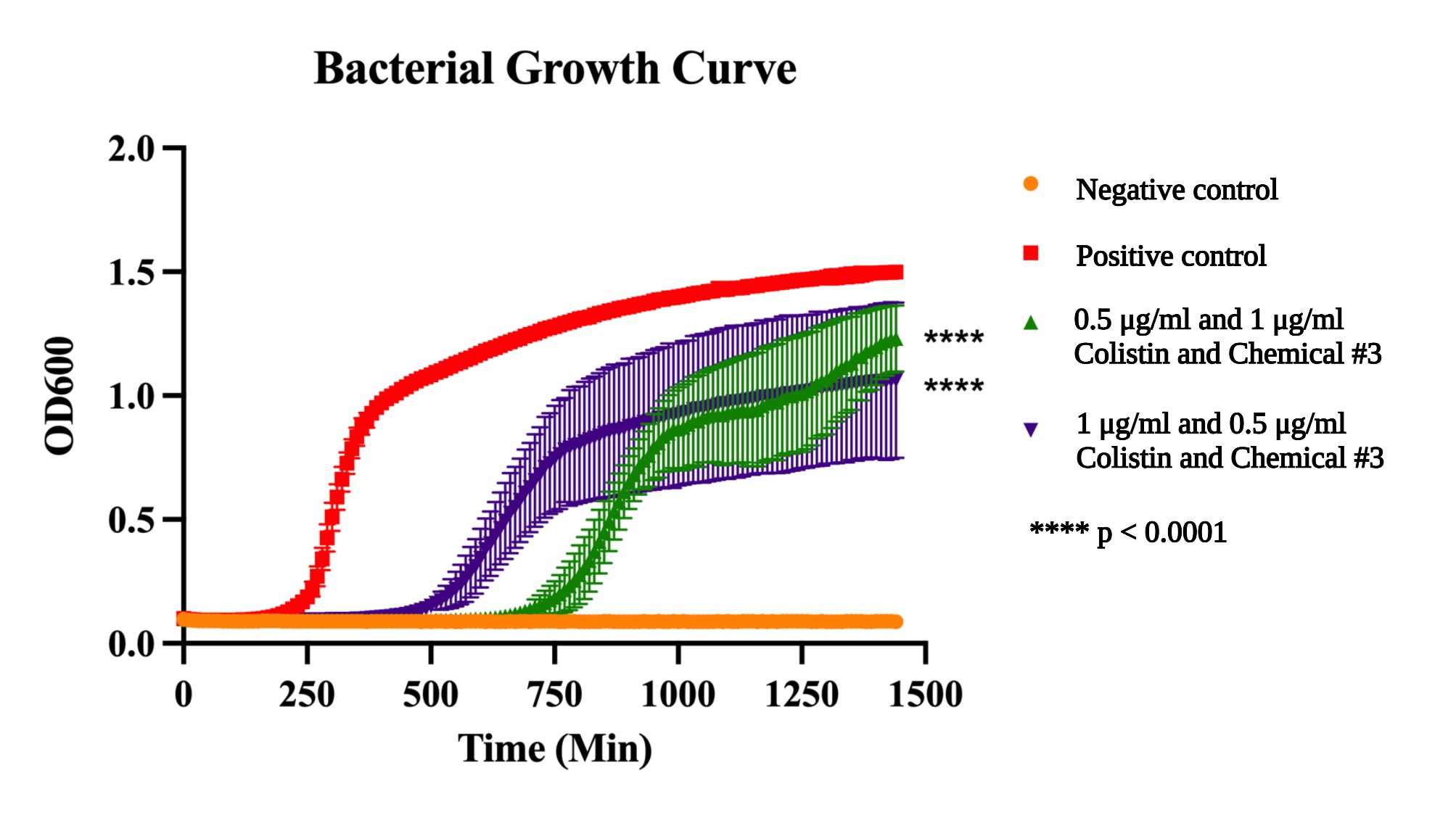


**Figure S12.** Bacterial growth curve for mid-log phase determination.
